# Multimodal intra-subject characterization of abdominal aortic aneurysm pathophysiology: a case study

**DOI:** 10.1007/s10237-026-02052-y

**Published:** 2026-03-29

**Authors:** Bahman Kargarbahrkhazar, Sanaz Farmani, Emma G. Foster, Rail Gilyazov, Sayed Ahmadreza Razian, Jason MacTaggart, Aditya N. Bade, Majid Jadidi

**Affiliations:** 1 Department of Biomechanics, University of Nebraska Omaha, Omaha, NE USA; 2 Department of Pharmacology and Experimental Neuroscience, University of Nebraska Medical Center, Omaha, NE USA; 3 Department of Surgery, University of Nebraska Medical Center, Omaha, NE USA

**Keywords:** Abdominal aortic aneurysm (AAA), Aortic remodeling, Matrix metalloproteinases (MMPs), Vascular biomechanics

## Abstract

Abdominal aortic aneurysm (AAA) is a degenerative vascular disease characterized by significant remodeling of the aortic wall. This study presents a comprehensive, multimodal analysis of AAA pathophysiology by comparing aneurysmal abdominal aortic tissue with non-aneurysmal thoracic aortic tissue of the same deceased human donor (N = 2), eliminating inter-subject differences. The multimodal approach integrated micro-CT imaging, transverse section histology, matrix metalloproteinase (MMP) analysis, and planar biaxial mechanical testing. Compared to the subject-matched thoracic controls, the AAA segments exhibited severe and heterogeneous degradation of the elastic network, profound loss of smooth muscle cells, extensive fibrosis, and a significant increase in calcification volume. Biochemically, AAA tissue showed elevated total MMP activity. In parallel, gelatin zymography tests validated an increase in MMP-9 activity in the diseased segments. Moreover, AAA tissues were substantially stiffer and less extensible than the corresponding healthy aortic tissue. These data, taken together, underscore the interconnected roles of elastin degradation, proteolytic enzyme activity, and fibrotic remodeling in the mechanical failure of the aortic wall, while highlighting the value of an intra-subject study design for elucidating disease-specific mechanisms.

## Introduction

Abdominal aortic aneurysm (AAA) is a pathological dilation of the abdominal aorta that carries a high risk of rupture when left untreated (Sakalihasan et al. 2018). It affects ~ 1–5% of the adult global population (Li et al. 2013). Prevalence rises sharply with age, reaching 4–8% of men and 0.5–2.2% of women over 60 years (Fleming et al. 2005; Guirguis-Blake et al. 2019). Histologically, AAA disease is characterized by degenerative remodeling of the aortic wall. Aneurysmal segments show significant elastin loss in the media along with a profound depletion of vascular smooth muscle cells (SMCs) (López-Candales et al. 1997; Bruijn et al. 2021). Another hallmark of AAA tissue is the presence of calcification within the lesion. High-resolution imaging and histology reveal scattered microcalcifications, and in some cases, sheet-like calcification throughout the degenerated media and intima of AAAs (Bruijn et al. 2021).

Matrix metalloproteinases (MMPs)—proteolytic enzymes capable of degrading extracellular matrix—play a central role in AAA development. The elastin fragmentation and collagen turnover in AAA walls have been linked to the overexpression of MMPs, especially the 72 kDa gelatinase MMP-2 and the 92 kDa gelatinase MMP-9 (Sakalihasan et al. 1996; Longo et al. 2002). Both MMP-2 and MMP-9 levels are markedly elevated in human AAA tissue compared to normal aortic wall (Sakalihasan et al. 1996; Longo et al. 2002). MMP-9, largely produced by infiltrating macrophages, is the most abundantly active elastase in aneurysmal tissue and can be detected at high levels in AAA wall (Thompson et al. 1995). MMP‑2 is constitutively produced by resident SMCs and macrophages; yet, its activity rises markedly in aneurysmal tissue, where it cleaves elastin and basement‑membrane collagen (Quintana and Taylor 2019). The concerted action of these and other proteases causes progressive degradation of elastic fibers and collagen, weakening the structural integrity of the aorta. The significant structural changes in AAA tissue alter the biomechanical properties of the aortic wall (Humphrey and Holzapfel 2012; Niestrawska et al. 2016). Planar biaxial testing of aneurysmal tissues has shown that they are often stiffer and less distensible than healthy aortas (Geest et al. 2004; Vande Geest et al. 2006).

Previous characterizations of AAA, while helped significantly in understanding the AAA pathophysiology, have been constrained by two major limitations. First, most studies rely on comparing diseased tissue from AAA patients with healthy tissue from different donors (Annabi et al. 2002; Geest et al. 2004; Vande Geest et al. 2006; Molacek et al. 2014; Niestrawska et al. 2016). This approach introduces inter-subject variability, confounding the results with differences in genetics, comorbidities, and baseline aortic properties (Hinterseher et al. 2011; Jadidi et al. 2020; Temprano‐Sagrera et al. 2024). Furthermore, prior human studies have typically focused on a single aspect of the disease, such as its mechanical properties, histological features, or enzymatic activity, with limited work combining these approaches. Therefore, an integrated approach is essential to correlate the complex interplay between the histopathological, biochemical, and biomechanical properties of aneurysmal tissue.

The present case study addresses these gaps by performing a comprehensive, multimodal analysis of entire aortas obtained from two donors with intact AAAs. By sampling both the aneurysmal abdominal segment and the non-aneurysmal thoracic segment from the same individual, each donor serves as their own control, effectively eliminating inter-subject variability. This approach, combining micro-CT imaging, transverse section histology, MMP analysis, and biaxial mechanical testing, allows for an integrated investigation into the pathological alterations specific to AAA disease and provides a more comprehensive view of its pathophysiology.

## Methods

### Tissue procurement and preparation

Intact aortas, extending from the descending thoracic aorta to the infrarenal abdominal aorta, were obtained from two female, deceased human donors (ages 64 and 89 years old) through an organ procurement organization, Live On Nebraska. The donors died of causes unrelated to aortic aneurysm rupture, and both aortas contained intact AAAs. All tissues were harvested within 24-h postmortem and were immediately transported to the laboratory in 0.9% phosphate-buffered saline (PBS) at 4 °C to maintain tissue viability. Upon arrival, aortas were stored at − 80 °C until processing. For this study, tissues were thawed at room temperature, and loose periadventitial tissue was carefully removed. For each donor, the non-aneurysmal descending thoracic aorta (TA) served as a patient-specific control for the abdominal aortic aneurysm (AAA) tissue. Orientation of the aorta (anterior vs. posterior) was maintained during dissection using anatomical landmarks, specifically the spinal column represented the posterior region, and the origins of the celiac and mesenteric arteries represented the anterior region.

### Gross photography and micro-computed tomography (Micro-CT) imaging

Following cleaning, each intact aorta was photographed to document gross morphology. To assess and quantify the distribution and volume of calcification, the aortas were imaged using a μCT Easy-TomS system (RX solutions, Chavanod, France). A 0.35-mm copper filter was used to reduce artifacts from calcification. The aorta from the 64-year-old donor was attached to a custom-made fixture, covered with plastic wrap to prevent dehydration, and pressurized to a near-physiological geometry with commercial shaving foam to open the lumen; shaving foam was used as its attenuation coefficient is invisible on X-ray. This aorta was scanned at 150 kV and 125 μA, with a frame rate of eight per projection and five projections per view, at a resolution of 97 μm. The aorta from the 89-year-old donor was scanned in its load-free state without pressure, at 100 kV and 350 μA with a frame rate of 10 frames per projection and four projections per view, at a resolution of 96 μm. Rotational raw TIFF images were converted to cross-sectional bitmaps and reconstructed using RX software (RX solutions, Chavanod, France). The cross-sectional images were then exported and compiled in Materialise Mimics v25 (Materialise NV, Leuven, Belgium) to create 3D models. Soft tissue and calcification were segmented and reconstructed separately, and the volume of calcium was recorded relative to the total tissue volume of the analyzed segment.

### Sample preparation for multimodal analysis

After imaging, specific regions of interest were identified and sampled from both the non-aneurysmal TA and the AAA segments of each aorta. For histological and biochemical analyses, several full-thickness rings (~ 2 mm in axial length) were excised. For mechanical analysis, a square specimen approximately 1 × 1 cm was cut from the anterior aspect of each region. All excised samples were kept hydrated in PBS at room temperature during preparation.

### Histology and immunohistochemistry

A full-thickness ring from both the TA and AAA regions of each donor was fixed in formalin, dehydrated through a graded ethanol series, and embedded in paraffin. The embedded rings were sectioned circumferentially at a thickness of 5 µm to allow for analysis of the extracellular matrix (ECM) around the vessel’s circumference. These transverse sections were stained with Movat’s Pentachrome to assess overall ECM structure and quantify glycosaminoglycans (GAGs), and Verhoeff-Van Gieson (VVG) for elastin. Masson’s Trichrome (MTC) stain was used to assess total collagen distribution and cellular components. This stain does not differentiate between specific collagen types. For immunohistochemistry (IHC), adjacent sections were stained for alpha-smooth muscle actin (*α*-SMA) to identify vascular SMCs, as well as for MMP-2 and MMP-9. The slides were scanned at 20× magnification (Leica Aperio CS2 scanner) and converted from whole slide SVS format to JPEG using custom-developed Histology Image Viewer and Converter software (Razian and Jadidi 2023). This comprehensive histological analysis allowed for the direct comparison of wall structure, composition, and cellularity between the non-aneurysmal and aneurysmal segments within the same individual.

### Planar biaxial mechanical testing

The 1 × 1 cm square specimens from the TA and AAA regions were subjected to planar biaxial testing to determine their passive mechanical behavior as previously described (Kazim et al. 2024; Shahbad et al. 2025). Tests were conducted using a CellScale BioTester equipped with 2.5 N load cells while the samples were immersed in 0.9% PBS solution at 37 °C. The longitudinal and circumferential directions of the tissue were aligned with the test axes. Graphite particles were sprinkled on the specimen surfaces to track deformations in the central region, minimizing edge effects (Fig. 18, Appendix). The testing protocol consisted of three main steps: maximum stretch estimation, preconditioning, and the primary test. First, maximum stretch limits were estimated for each direction by subjecting the tissue to equibiaxial loading and unloading up to 1000 mN. Subsequently, during preconditioning, each specimen was stretched equibiaxially to these maximum stretches through 20 loading and unloading cycles to establish a repeatable pseudoelastic response. Following preconditioning, tissues were stretched equibiaxially to the maximum stretch levels, and the stress-stretch response was determined to compare the mechanical behavior between the TA and AAA regions. All tests were performed at a stretch rate of 1% per second, and data and images were acquired at 5 Hz. Assuming incompressibility, the experimental Cauchy stresses were calculated from the applied loads and measured deformations.

### Matrix metalloproteinase (MMP) analysis

Tissue samples collected from the TA and AAA sections were snap-frozen and stored at − 80 °C for MMP analysis. Tissues were homogenized in NP-40 lysis buffer using a Qiagen TissueLyzer II (Valencia, CA). Lysates were centrifuged at 16,000 g at 4 °C for 20 min and supernatants were collected and stored at − 80 °C for further analysis. Total protein in each sample was quantitated using the Pierce™ BCA Protein Assay Kit (Thermo Fisher Scientific, Waltham, MA).

Gelatin zymography was performed to assess MMP-2 and MMP-9 activities in TA and AAA tissue lysates as previously described (Bade et al. 2021; Foster et al. 2023). In this assay, 10 μg of protein for each sample was loaded in a 10% SDS–polyacrylamide gel containing 0.1% gelatin and gels were run at 55 V. Recombinant human MMP-2 and MMP-9 were used as standards. After the completion of a run, gels were removed and washed with water for 20 min followed by incubation with renaturation buffer [2.5% (v/v) Triton X-100 in Milli-Q water] for 90 min at room temperature on a shaker. Next, gels were incubated in developing buffer (50 mM Tris–HCl, pH 7.5, 5 mM CaCl_2_, 0.2 M NaCl, and 0.02% Brij-35) for 48 h at 37 °C in a shaker (Innova 42, New Brunswick Scientific, Edison, NJ). Afterward, gels were washed with water for 30 min and later stained with 0.2% Coomassie Brilliant Blue R-250 (BIO-RAD, Hercules, CA) for 1 h. After staining, gels were washed with water for 30 min followed by a wash with a destaining solution (30% methanol, 10% acetic acid, and 60% water) for 45 min. Later, gels were washed with water for 20 min to remove any destaining solution before being stored in fresh water until imaging. The stained gels were imaged using the iBright 750 Imaging System (Invitrogen, Carlsbad, CA).

Broad-spectrum MMP activity was measured in TA and AAA tissue lysates using a quenched fluorogenic substrate, OMNIMMP® RED (BML-P277-0100, Enzo Life Sciences) as previously described (Bade et al. 2021). The composition of the quenched fluorogenic substrate was TQ3-GABA-Pro-Cha-Abu-Smc-His-Ala-Dab (6’-TAMRA)-Ala-Lys-NH2 [TQ3 = quencher; GABA = 4-aminobutyric acid; Cha = L-cyclohexylalanine; Abu = 2-aminobutyric acid; Smc = S-methyl-L-cysteine; Dab = 2,4-diaminobutyric acid; 6’-TAMRA = 6’-tetramethylrhodamine]. This assay was performed in a 96-well, black, ½ well microplate format. In each well, 50 μg of protein from each tissue lysate was incubated with a buffer (50 mM HEPES, pH 7.5, 10 mM CaCl_2_, and 0.05% Brij-35) at 37 °C for 60 min. Each sample was plated in duplicate. After incubation, OMNIMMP® RED substrate was added to each well to start the reaction. The plate was continuously read at Ex/Em = 545/576 nm (cutoff at 570 nm) for 300 min with 5-min time intervals at constant temperature, 37 °C, using Molecular Devices SpectraMax M3 plate reader with SoftMax Pro 6.2 software. Broad-spectrum MMP activity was measured as the change in fluorescence intensity (RFU) per minute per μg protein (RFU/min/μg of protein).

## Results

### Micro-CT analysis of aortic calcification

**Fig. 1:**
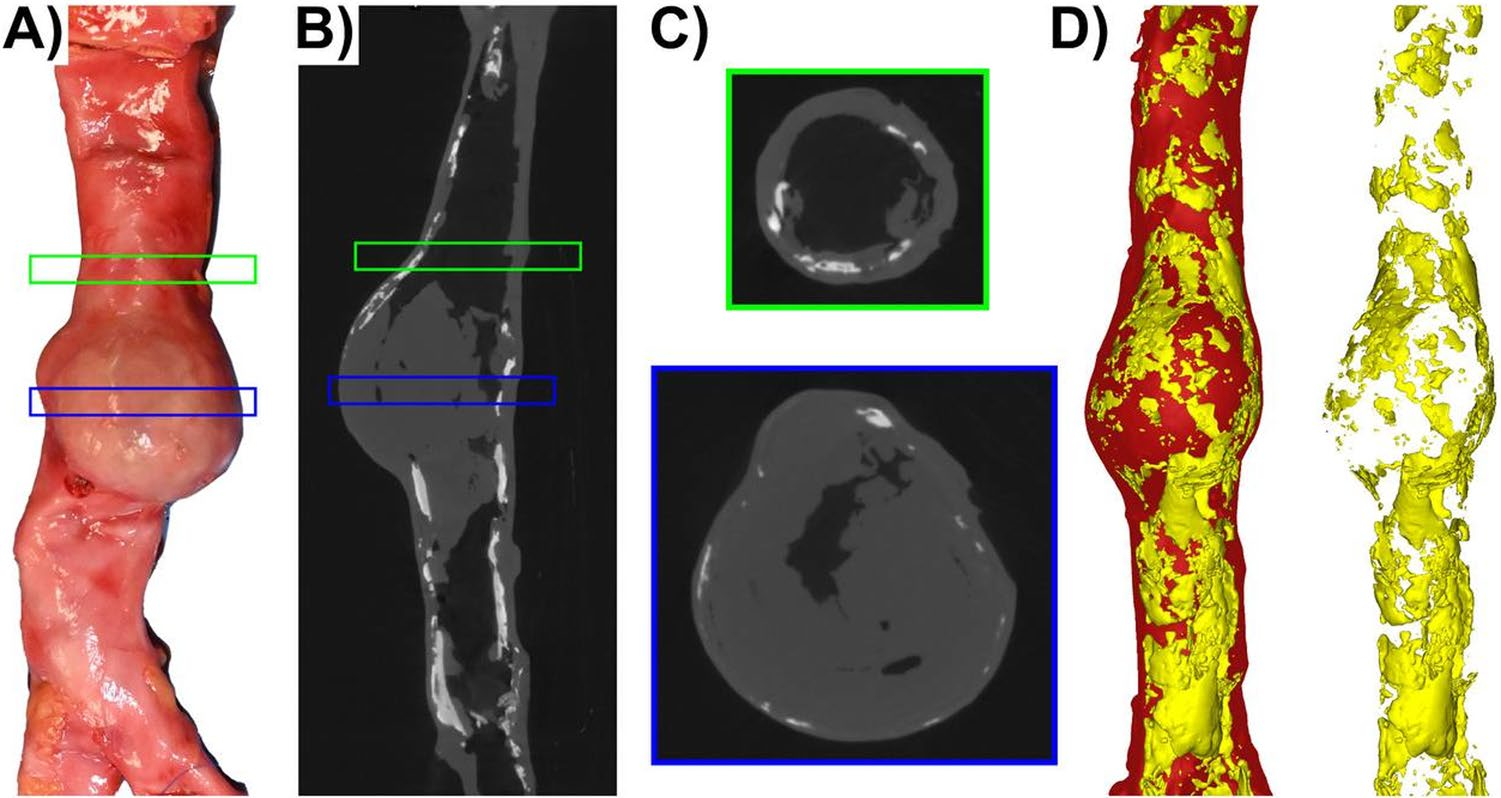
Multimodal imaging of the aorta from the 64-year-old female donor with an intact AAA. The aorta was pressurized with shaving foam prior to micro-CT imaging to achieve a near-physiological geometry. From left to right: **A** Gross photograph of the whole aorta; **B** Longitudinal micro-CT view showing calcified plaques; **C** Representative cross-sectional micro-CT images from the non-aneurysmal thoracic aorta (top, green box) and the AAA segment (bottom, blue box); **D** A 3D reconstruction of the aorta (red) with calcification (yellow); and an isolated 3D reconstruction of the calcification, showing its extensive sheet-like and nodular distribution

Micro-CT imaging showed extensive calcification in the aortas from both donors, with distinct patterns and distributions associated with the aneurysmal segments. In the 64-year-old donor (Fig. 1), the total calcification volume was substantial throughout the aorta. Quantitative analysis measured a calcification volume of 247.88 mm^3^ in the thoracic segment 21-mm proximal to the AAA; within the 35-mm-long AAA segment itself, the volume was 512.96 mm^3^, and it decreased to 491.90 mm^3^ in the segment 21-mm distal to the aneurysm. Morphologically, the calcification was predominantly sheet-like along the length of the aorta. However, within the aneurysmal bulge, fragmented sheets were interspersed with calcification nodules. The mean calcification thickness across the anterior and posterior walls proximal to the AAA was 1.22 mm, 0.74 mm within the AAA segment, and 1.46 mm for the distal part to the AAA.

**Fig. 2:**
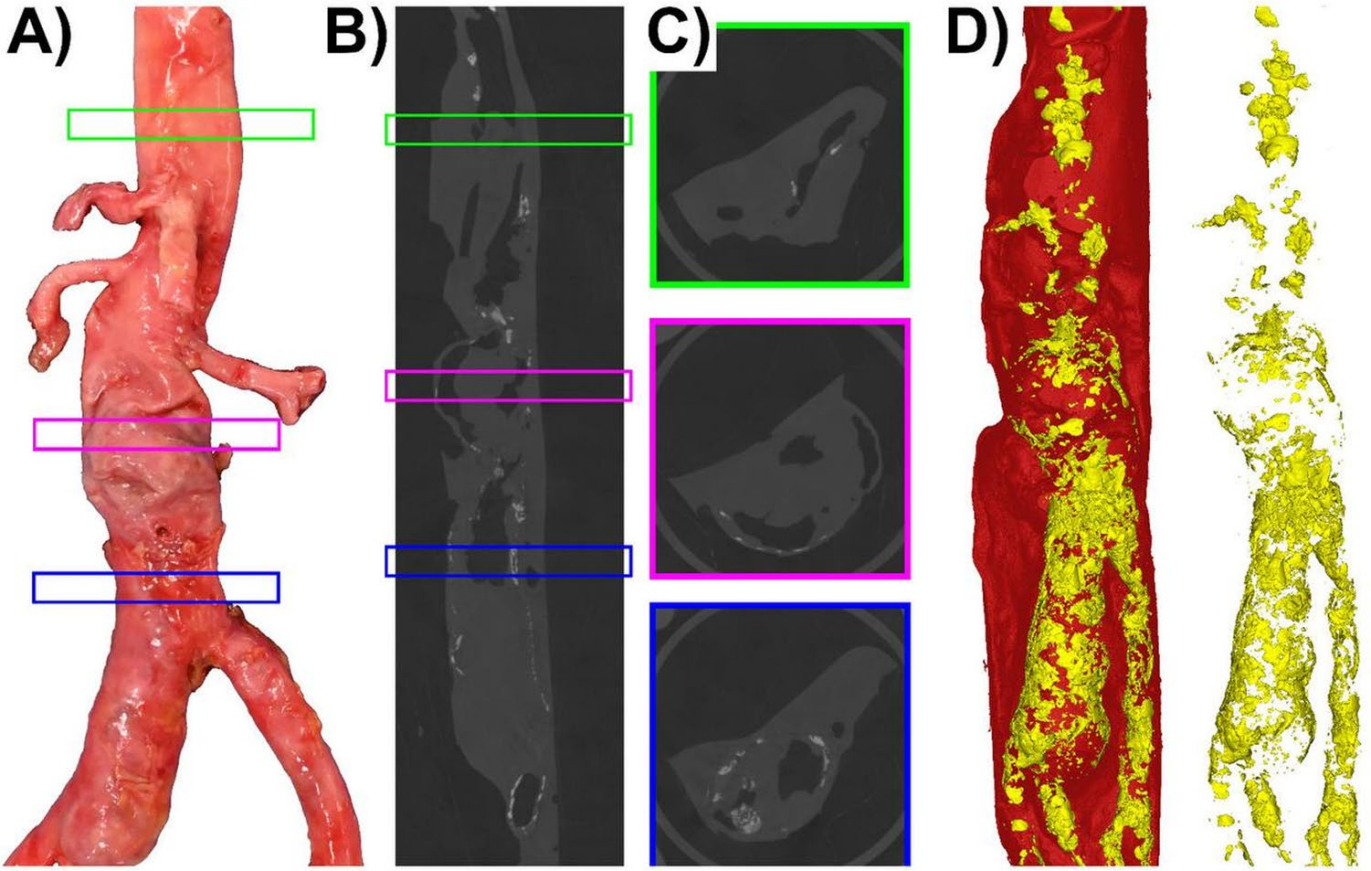
Multimodal imaging of the aorta from the 89-year-old female donor with an AAA extending into the iliac arteries. This aorta was imaged with micro-CT in its collapsed, non-pressurized state. From left to right: **A** Gross photograph of the aorta; **B** Longitudinal micro-CT view; **C** Representative cross-sectional micro-CT images from proximal (green box), mid-aneurysm (pink box), and distal (blue box) segments; **D** A 3D reconstruction of the aorta (red) showing the distribution of calcification (yellow); and an isolated 3D view of the calcification, highlighting the sheet-like and nodular morphology

The aorta from the 89-year-old donor showed a complex aneurysm that extended into the right common iliac artery (Fig. 2). The calcification volume measured 190.55 mm^3^ at 21-mm proximal to the AAA, 565.42 mm^3^ within the 42-mm-long AAA segment, and a significantly larger volume of 1417.26 mm^3^ in the 21-mm distal segment. The calcification morphology was also predominantly sheet-like. Notably, fragmented and thinner calcium sheets were observed in the iliac portion of the aneurysm, while distinct nodular calcifications were present proximal to the aortic bifurcation. The mean calcification thickness across the anterior and posterior walls proximal to the AAA was 1.32 mm, 0.59 mm within the AAA segment, and 1.36 mm for the distal part to the AAA.

### Histological and immunohistochemical analysis

#### Aorta from 64-year-old donor

**Fig. 3:**
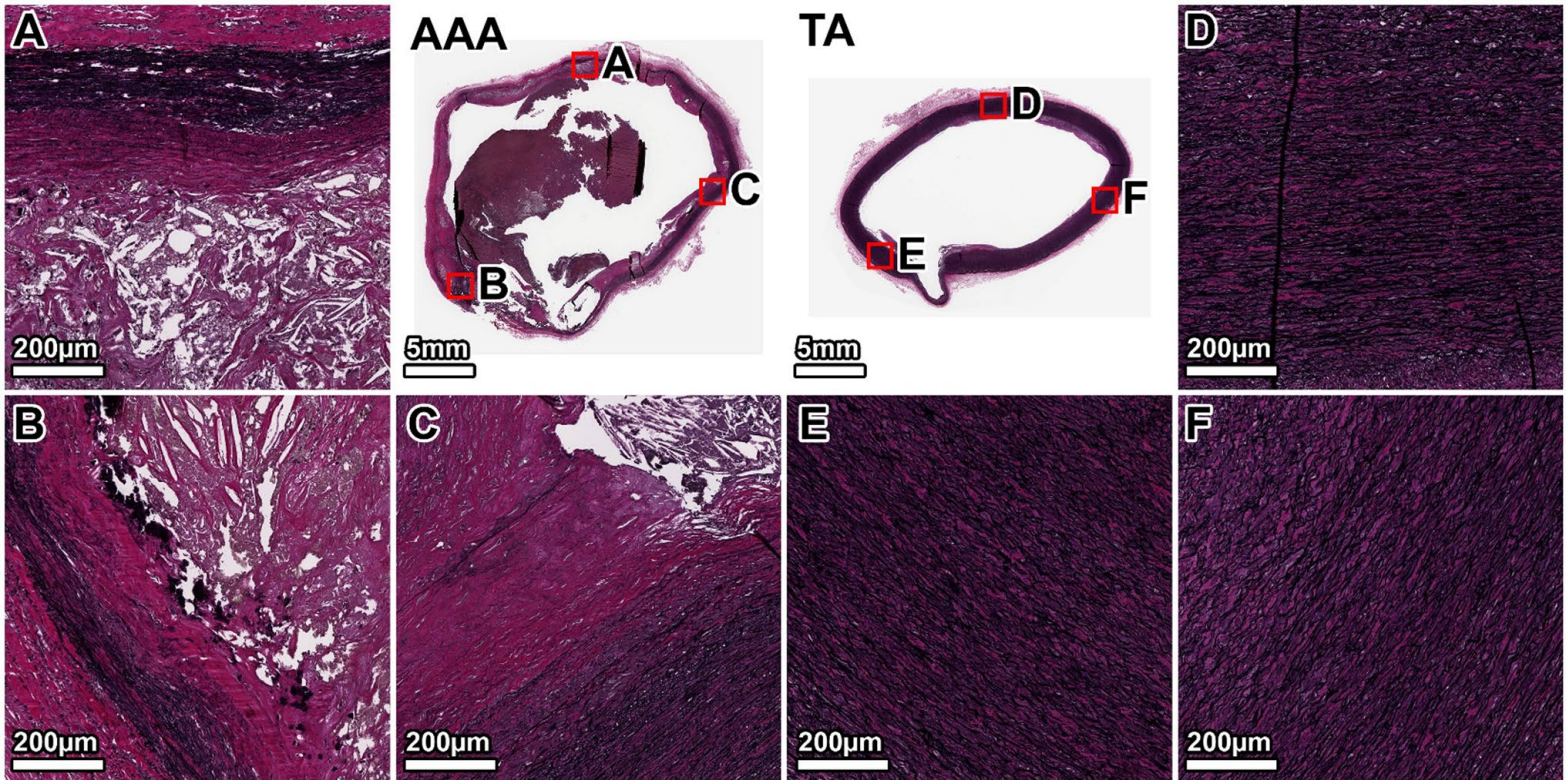
Elastin architecture in the 64-year-old donor. VVG staining (elastin: black) shows severe, heterogeneous elastin degradation in the AAA wall compared to the organized, dense lamellae of the TA. Panels show whole-section views of the AAA and TA rings, with high-magnification views from the lateral (**A**, **D**), anterior (**B**, **E**), and posterior (**C**, **F**) walls. The artifactual disruption seen in the anterior wall of the TA ring was caused by a large calcium deposit that made sectioning with the microtome difficult, even after surface decalcification

VVG staining of the TA demonstrated a healthy elastic artery structure, with uniform, thick, and tightly packed elastic lamellae throughout the media (Fig. 3, panels D–F). In contrast, the AAA wall showed severe and heterogeneous degradation of the elastic network. The anterior wall exhibited the most profound disruption, with a near-total loss of lamellar architecture (Fig. 3, panel B). The lateral and posterior walls also displayed significant elastin fragmentation and thinning, with some disorganized elastic remnants visible in the outer media (Fig. 3, panels A, C). A large mural thrombus was present, adhering to the anterior aspect.

**Fig. 4:**
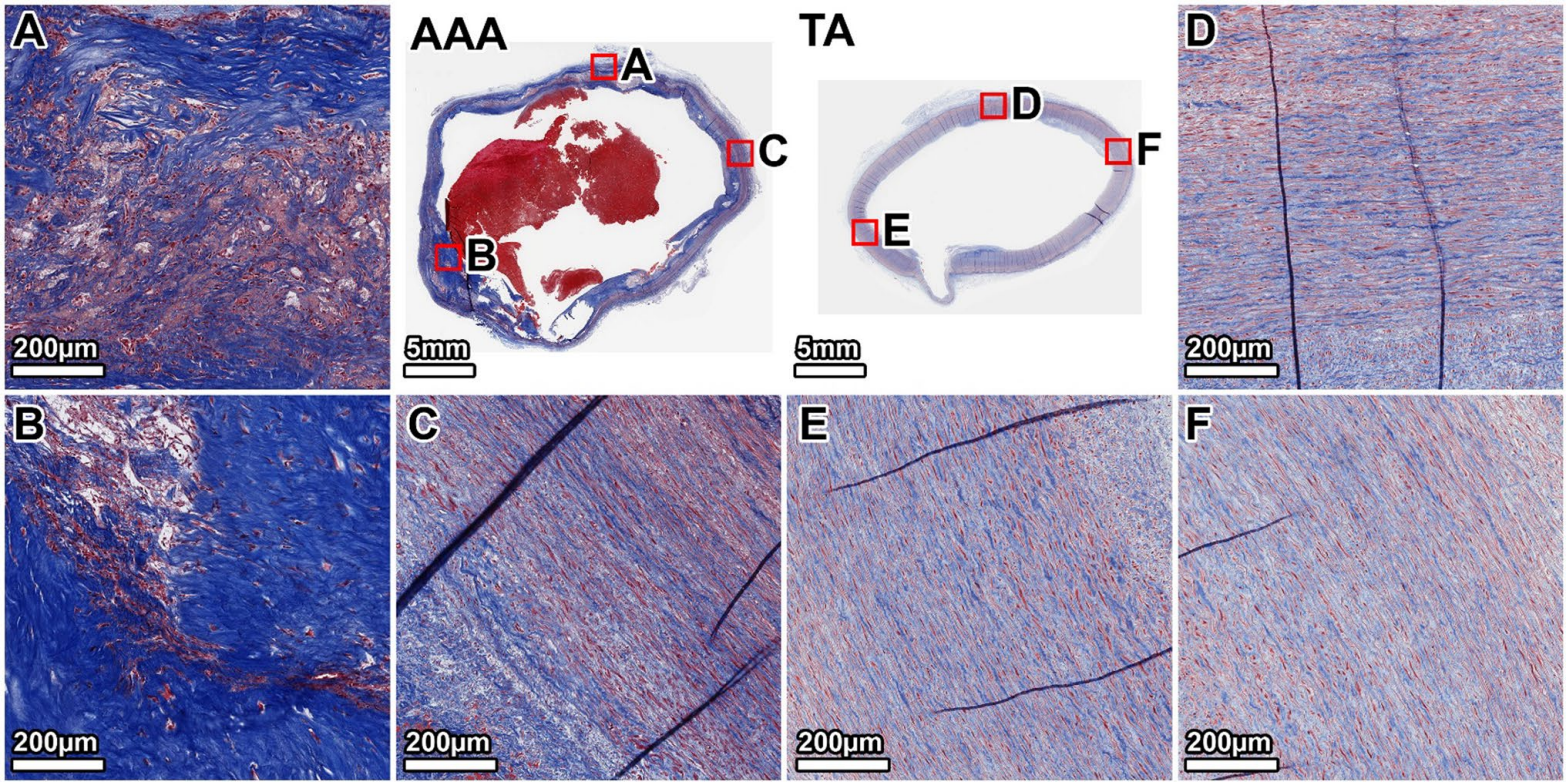
Collagen deposition in the 64-year-old donor. MTC staining (collagen: blue) reveals extensive fibrosis throughout the AAA media, which is largely absent in the TA. The fibrotic remodeling is most pronounced in the anterior wall. Panels show whole-section views and high-magnification views from the lateral (**A**, **D**), anterior (**B**, **E**), and posterior (**C**, **F**) walls. The artifactual disruption seen in the anterior wall of the TA ring was caused by a large calcium deposit that made sectioning with the microtome difficult, even after surface decalcification

MTC staining of the TA showed minimal collagen (blue) within the tunica media, which appeared healthy without noticeable fibrosis (Fig. 4, panels D–F). The AAA wall, however, was characterized by extensive fibrosis. Dense deposits of collagen were observed throughout the media, replacing the degraded elastic lamellae. This fibrotic response was most intense in the anterior wall, corresponding to the region of greatest elastin loss (Fig. 4, panel B), while the lateral and posterior walls also showed significant, though slightly less severe, collagen infiltration (Fig. 4, panels A, C).

**Fig. 5:**
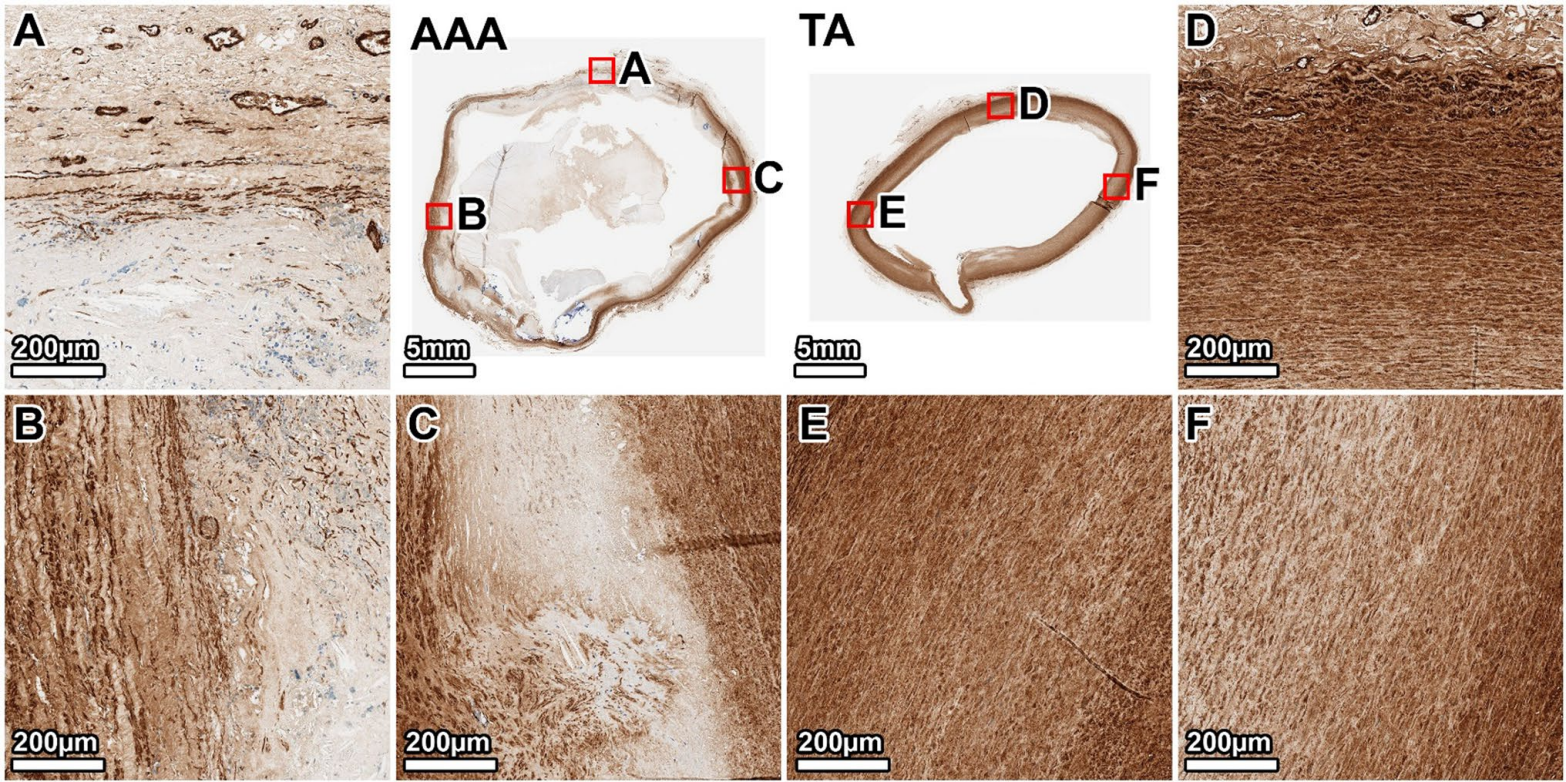
Smooth muscle cell distribution in the 64-year-old donor. *α*-SMA staining (SMCs: brown) demonstrates a profound and heterogeneous depletion of SMCs in the AAA wall compared to the dense, organized SMC population in the TA. Panels show whole-section views and high-magnification views from the lateral (**A**, **D**), anterior (**B**, **E**), and posterior (**C**, **F**) walls. The artifactual disruption seen in the anterior wall of the TA ring was caused by a large calcium deposit that made sectioning with the microtome difficult, even after surface decalcification

Staining for *α*-SMA confirmed a dense and uniformly distributed population of SMCs (brown) throughout the TA media (Fig. 5, panels D–F). The AAA wall showed a significant and widespread loss of SMCs. This depletion was heterogeneous; the anterior and lateral walls showed a near-complete loss, particularly in the inner media (Fig. 5, panels B, A). The posterior wall retained a slightly higher, albeit sparse and disorganized, population of SMCs, primarily in the outer media where some elastic lamellae persisted (Fig. 5, panel C).

**Fig. 6:**
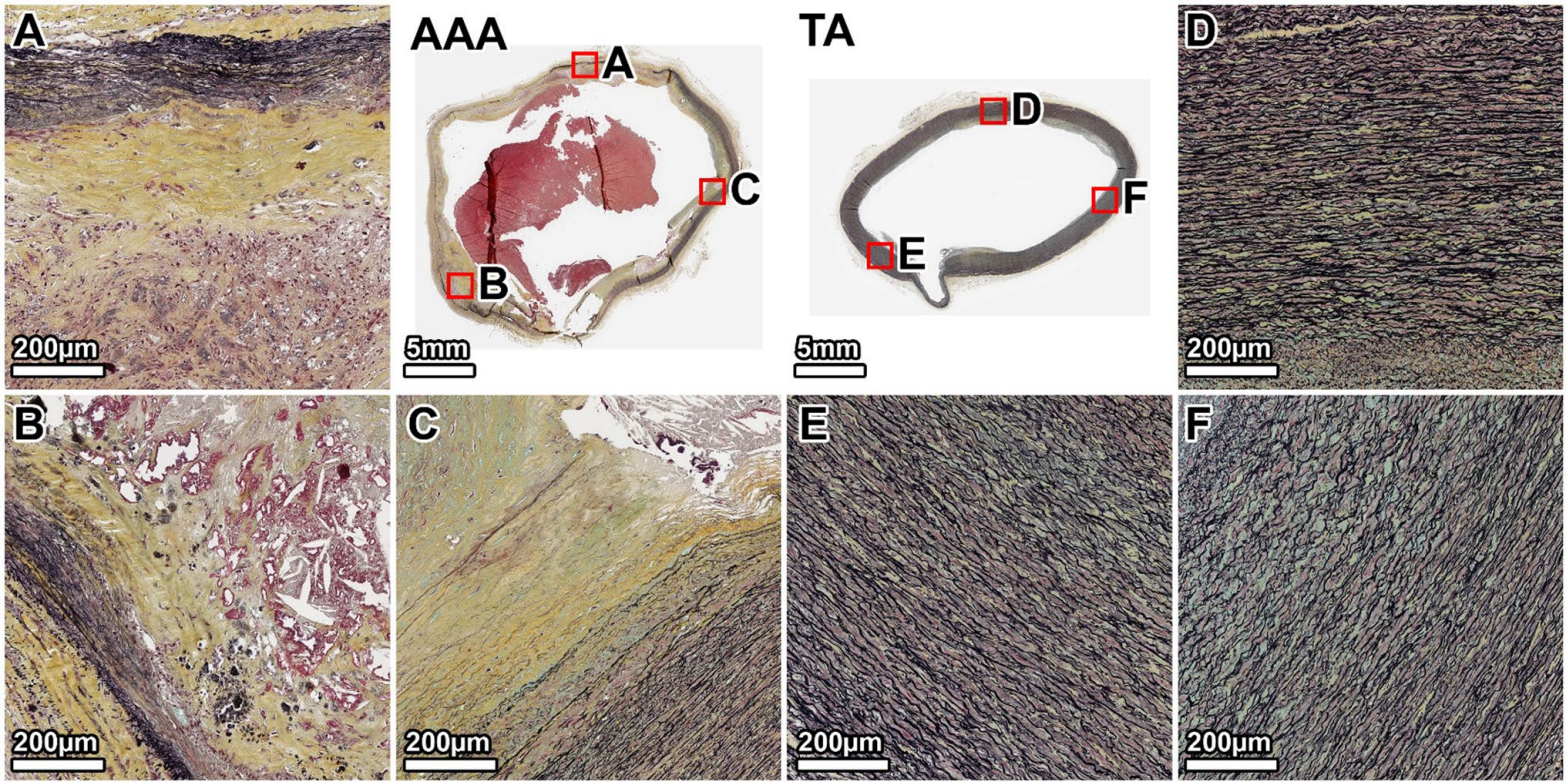
Overall ECM remodeling in the 64-year-old donor. Movat’s Pentachrome stain (elastin: black; collagen: yellow; and GAGs: gray-green) highlights the disorganized, pathological matrix of the AAA, characterized by elastin loss, fibrosis, and GAG accumulation, in contrast to the structured TA wall. Panels show whole-section views and high-magnification views from the lateral (**A**, **D**), anterior (**B**, **E**), and posterior (**C**, **F**) walls. The artifactual disruption seen in the anterior wall of the TA ring was caused by a large calcium deposit that made sectioning with the microtome difficult, even after surface decalcification

Movat’s Pentachrome staining provided an overview of the ECM remodeling. The TA exhibited a highly organized structure with clearly defined lamellar units of elastin (black) (Fig. 6, panels D–F). The AAA wall was completely disorganized, showing significant accumulation of GAGs (gray-green), extensive collagen deposition (yellow), and fragmented elastin remnants. These pathological changes were evident circumferentially but were most severe in the anterior wall at the interface with the mural thrombus (Fig. 6, panel B).

#### Aorta from 89-year-old donor

**Fig. 7:**
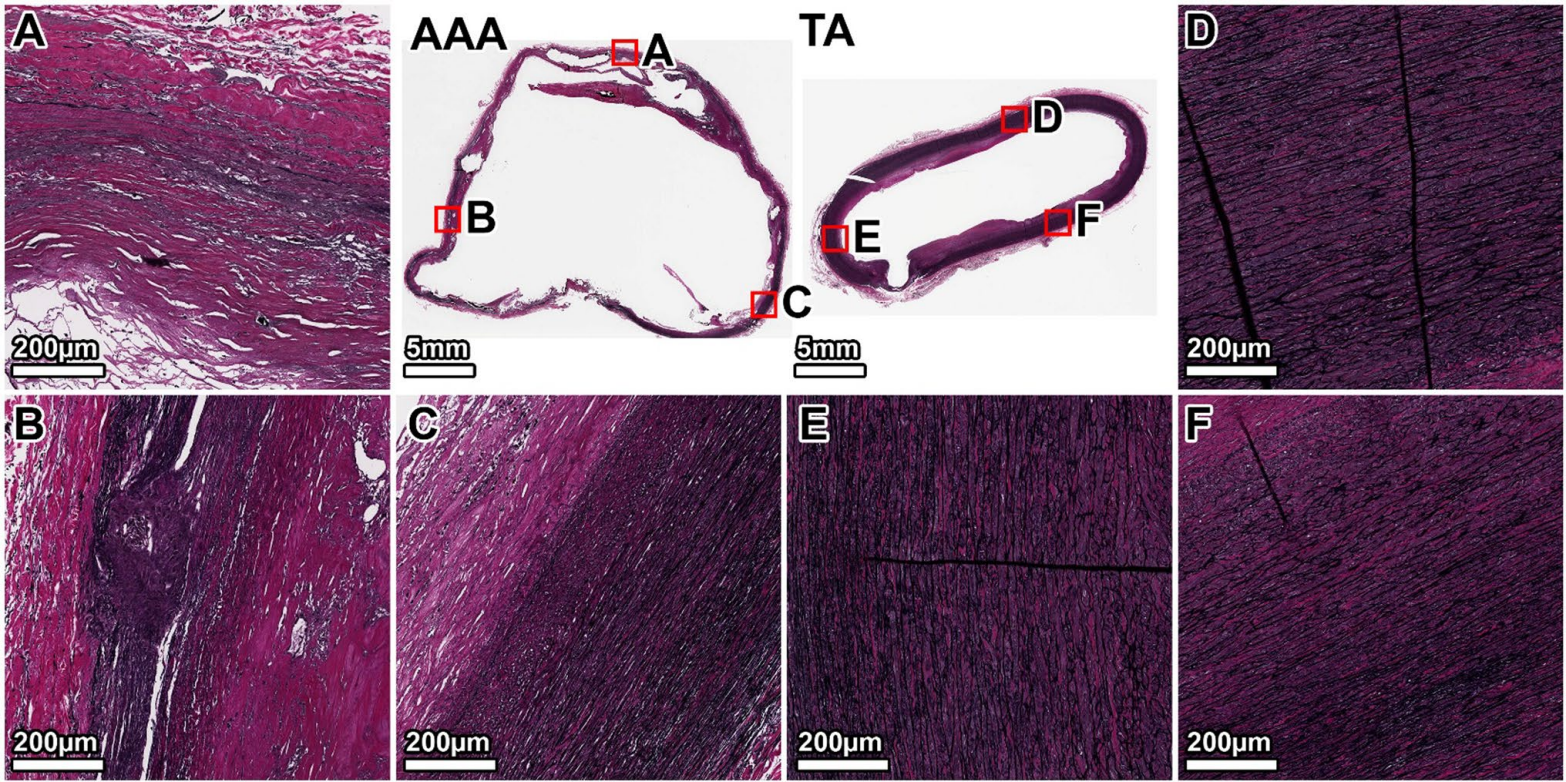
Elastin architecture in the 89-year-old donor. VVG staining shows a near-total, circumferential loss of medial elastin in the AAA. The TA displays age-related thinning of lamellae but remains structurally intact. Panels show whole-section views and high-magnification views from the posterior (**A**, **D**), right anterolateral (**B**, **E**), and left anterolateral (**C**, **F**) walls. The artifactual disruption seen in the anterior wall of the TA ring was caused by a large calcium deposit that made sectioning with the microtome difficult, even after surface decalcification

The TA from the 89-year-old donor showed age-related changes, including thinner and more widely spaced elastic lamellae, but the overall architecture remained intact (Fig. 7, D–F panels). The AAA wall, by comparison, exhibited a significant and heterogeneous loss of elastin. The degradation was most severe in the posterior and right anterolateral walls, which were almost entirely devoid of organized elastic structures (Fig. 7, panels A, B). In contrast, the left anterolateral wall retained significantly more intact elastic lamellae, particularly in the outer portion of the tunica media (Fig. 7, panel C).

**Fig. 8:**
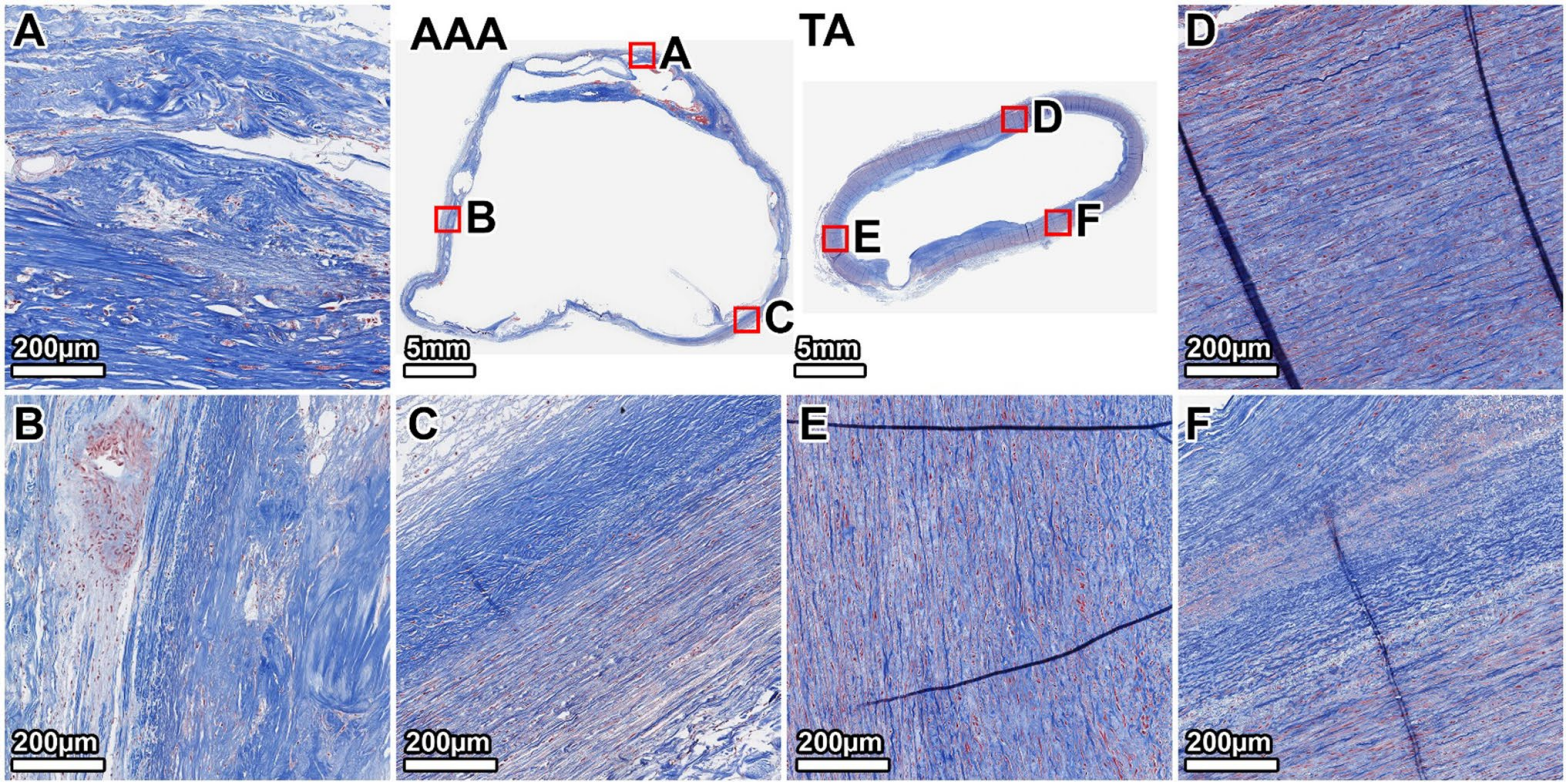
Collagen deposition in the 89-year-old donor. MTC staining reveals a heavily fibrotic and largely acellular AAA wall. This contrasts with the organized, cellular media of the TA. Panels show whole-section views and high-magnification views from the posterior (**A**, **D**), right anterolateral (**B**, **E**), and left anterolateral (**C**, **F**) walls. The artifactual disruption seen in the anterior wall of the TA ring was caused by a large calcium deposit that made sectioning with the microtome difficult, even after surface decalcification

MTC staining of the TA showed minimal collagen within the media, but more than the younger donor (Fig. 8, panels D–F). The AAA wall was almost completely replaced by dense, acellular collagen. The fibrosis was heterogeneous; the posterior and right anterolateral walls showed the most intense collagen deposition (Fig. 8, panels A, B). The left anterolateral wall, which corresponded to the area of least elastin degradation, exhibited comparatively less fibrosis (Fig. 8, panel C).

**Fig. 9:**
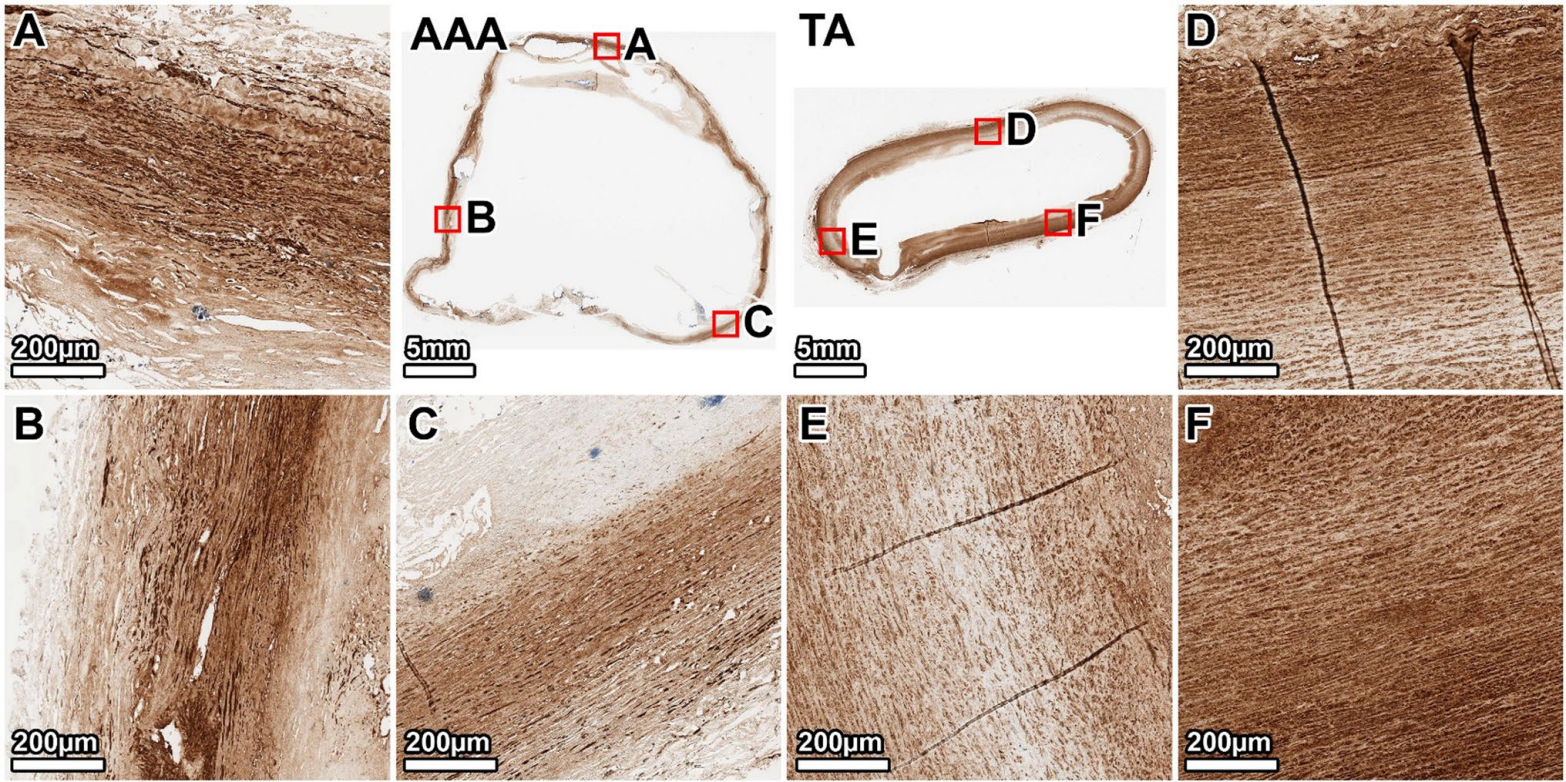
Cellular composition in the 89-year-old donor. *α*-SMA staining reveals a large population of *α*-SMA-positive cells, likely myofibroblasts, in the fibrotic regions. The TA shows a uniform distribution of SMCs. Panels show whole-section views and high-magnification views from the posterior (**A**, **D**), right anterolateral (**B**, **E**), and left anterolateral (**C**, **F**) walls. The artifactual disruption seen in the anterior wall of the TA ring was caused by a large calcium deposit that made sectioning with the microtome difficult, even after surface decalcification

*α*-SMA staining in the TA showed a reduced density of SMCs compared to the younger donor, consistent with aging, but the cells were still uniformly distributed (Fig. 9, panels D–F). In the AAA, the medial layer was largely devoid of the organized SMCs seen in the TA. However, a substantial population of *α*-SMA-positive cells was present, particularly in the heavily fibrotic posterior and right anterolateral walls (Fig. 9, panels A, B). Given the acellular, scar-like appearance of the wall on MTC staining, these cells likely represent myofibroblasts actively involved in the extensive fibrotic remodeling of the aneurysmal tissue rather than residual medial SMCs.

**Fig. 10:**
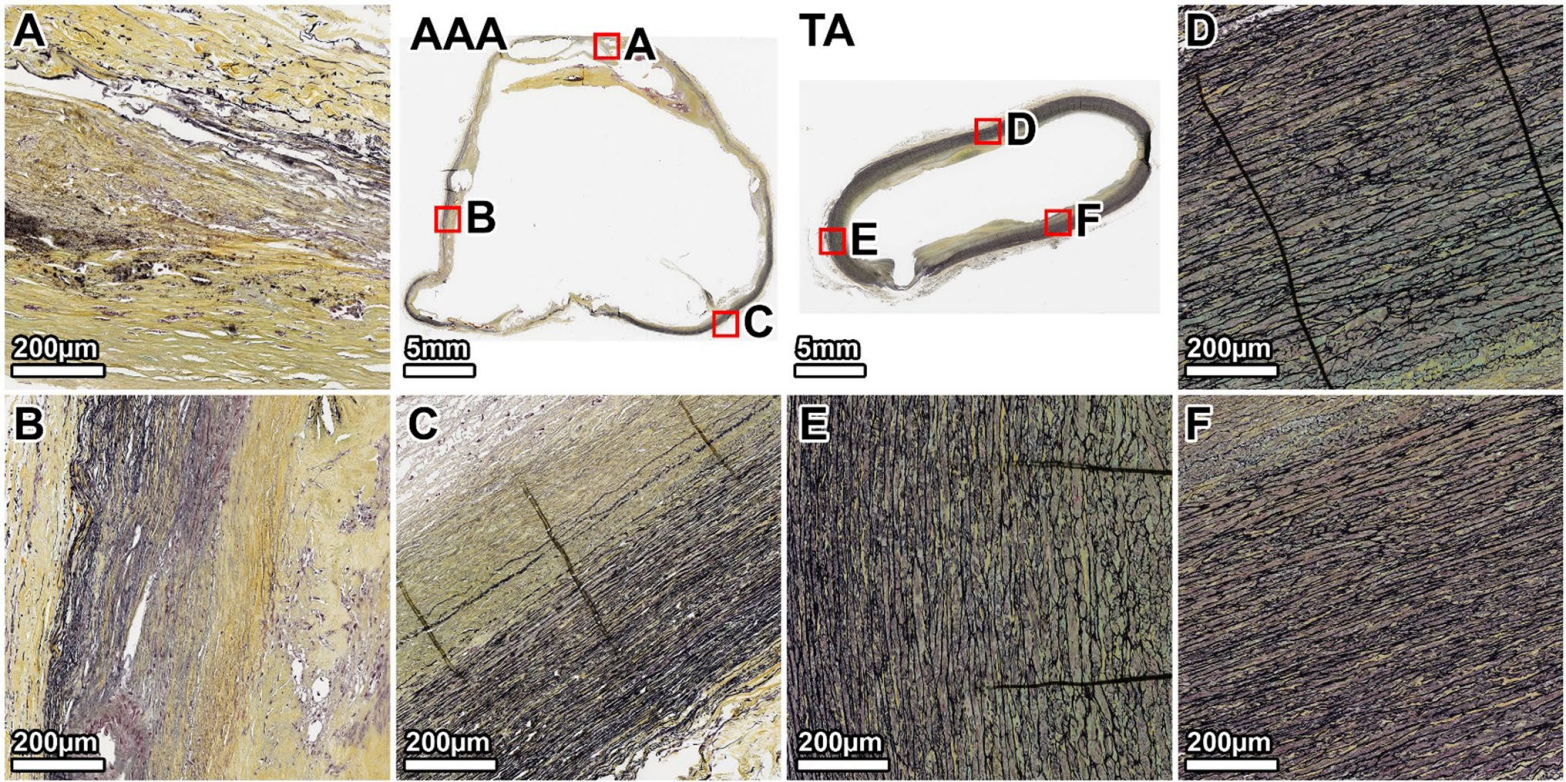
Overall ECM remodeling in the 89-year-old donor. Movat’s Pentachrome stain illustrates the end-stage pathology of the AAA wall, which is composed almost entirely of disorganized collagen and GAGs with a near-complete loss of elastin and cellularity. Panels show whole-section views and high-magnification views from the posterior (**A**, **D**), right anterolateral (**B**, **E**), and left anterolateral (**C**, **F**) walls. The artifactual disruption seen in the anterior wall of the TA ring was caused by a large calcium deposit that made sectioning with the microtome difficult, even after surface decalcification

Movat’s Pentachrome staining contrasted the organized TA wall with the pathological AAA matrix (Fig. 10). The AAA wall was a disorganized composite of dense collagen (yellow) and abundant GAGs (blue-green), with minimal residual elastin. This pathological composition varied circumferentially; the posterior and right anterolateral walls showed the most advanced degradation, appearing as dense, fibrotic tissue with significant GAG accumulation (Fig. 10, panels A, B). The left anterolateral wall, however, appeared less remodeled, retaining more of its underlying lamellar structure, consistent with the greater preservation of elastin in this region (Fig. 10, panel C).

### MMP expression

#### Aorta from 64-year-old donor

**Fig. 11:**
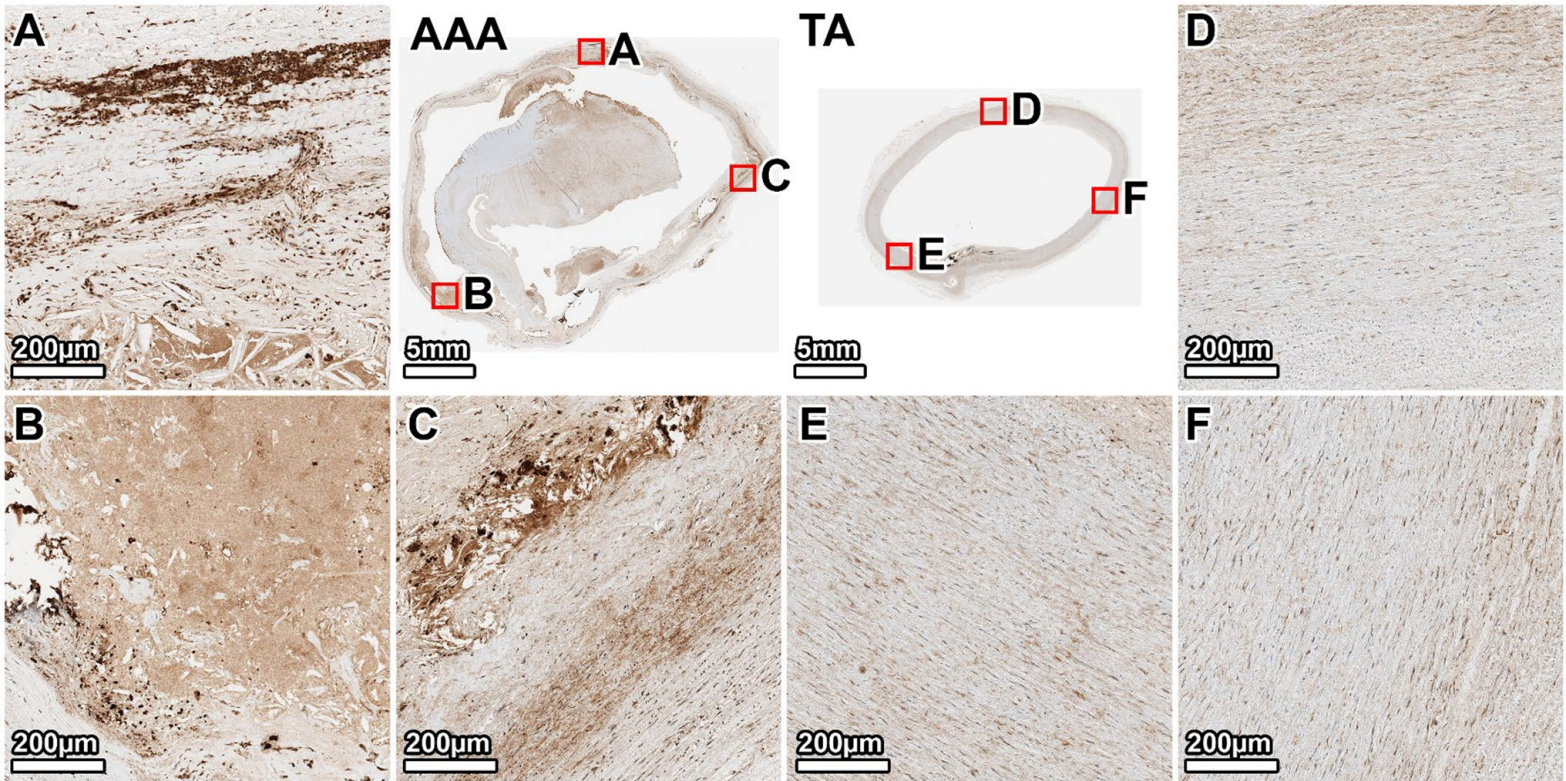
MMP-2 expression in the 64-year-old donor. Immunohistochemical staining for MMP-2 (brown) shows low, diffuse expression in the TA media. In the AAA, MMP-2 is intensely expressed at the thrombus–wall interface and in the tunica media. Panels show whole-section views of the AAA and TA rings, with high-magnification views from the lateral (**A**, **D**), anterior (**B**, **E**), and posterior (**C**, **F**) walls

In the TA, MMP-2 expression was low and diffusely present throughout the media (Fig. 11, panels D–F). In the AAA, MMP-2 staining was markedly increased and heterogeneously distributed. The most intense signal was localized to the border between the mural thrombus and the arterial wall, particularly in the anterior region where the thrombus was adherent (Fig. 11, panel B). The lateral and posterior walls also showed increased MMP-2 expressions in the tunica media (Fig. 11, panels A, C).

**Fig. 12:**
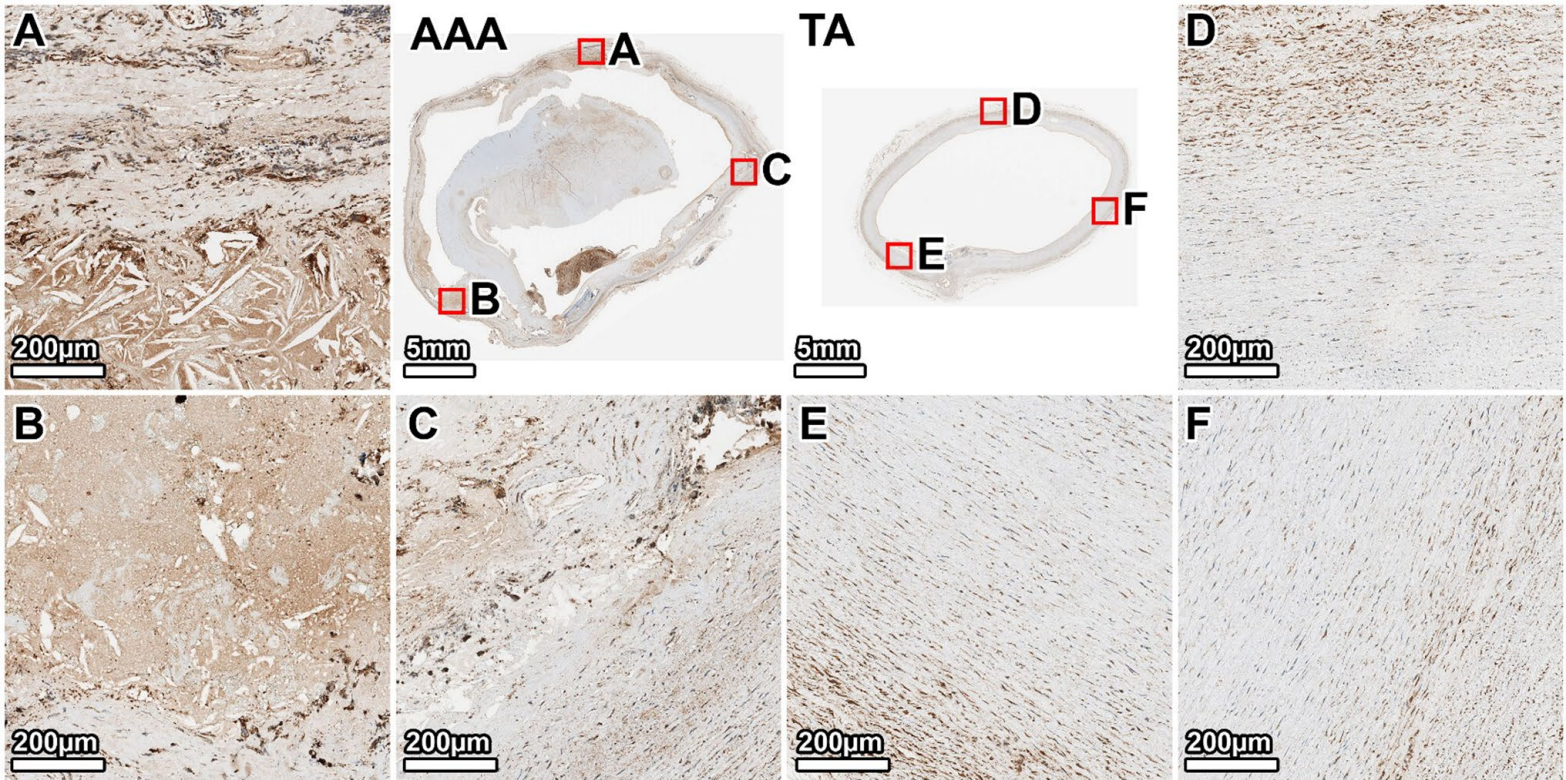
MMP-9 expression in the 64-year-old donor. Immunohistochemical staining for MMP-9 (brown) is faint and uniform in the TA. In the AAA, expression is heterogeneous, with a notable increase in the tunica media of the more preserved posterior and lateral wall regions. Panels show whole-section views of the AAA and TA rings, with high-magnification views from the lateral (**A**, **D**), anterior (**B**, **E**), and posterior (**C**, **F**) walls

MMP-9 expression in the TA was faint and evenly distributed, with slightly more density in the outer media (Fig. 12, panels D–F). In contrast, the AAA exhibited a non-uniform and more intense MMP-9 signal. In the preserved posterior wall, medial MMP-9 staining was relatively weak (Fig. 12, panel C). However, in the structurally more degraded anterior and lateral walls, MMP-9 was detectable in the tunica media (Fig. 12, panels B, A).

#### Aorta from 89-year-old donor

**Fig. 13:**
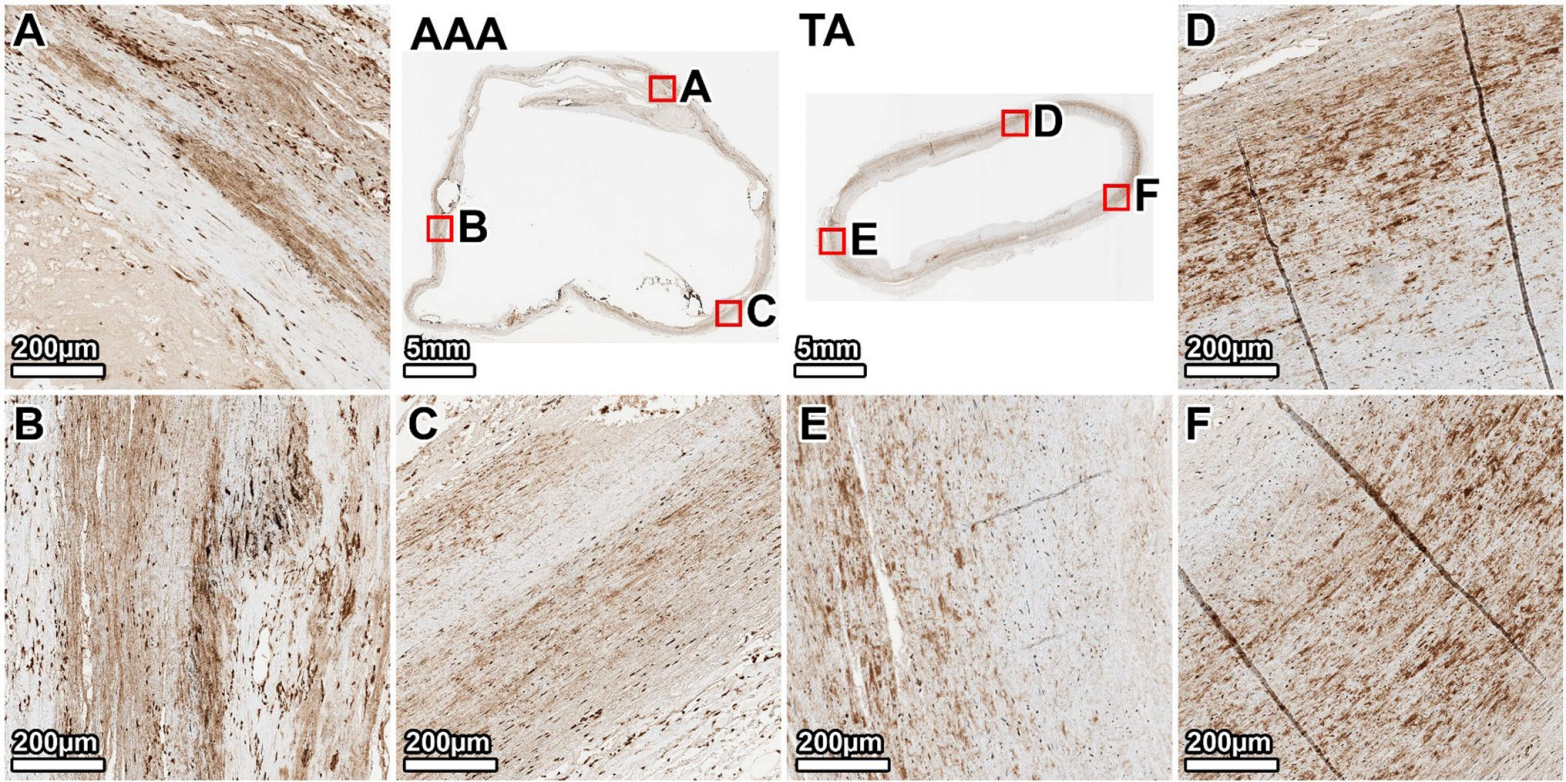
MMP-2 expression in the 89-year-old donor. Staining for MMP-2 is diffusely present in the aged TA. In the AAA, strong expression is co-localized with myofibroblasts in the extensively remodeled right anterolateral and posterior walls. Panels show whole-section views of the AAA and TA rings, with high-magnification views from the posterior (**A**, **D**), right anterolateral (**B**, **E**), and left anterolateral (**C**, **F**) walls

The TA of the older donor showed a diffuse, patchy MMP-2 signal throughout the media, consistent with age-related remodeling (Fig. 13, panels D–F). The fibrotic AAA wall displayed a strong and widespread MMP-2 signal. Expression was co-localized with the abundant myofibroblasts in the heavily remodeled posterior and right anterolateral walls (Fig. 13, panels A, B). The left anterolateral wall, which had greater elastin preservation and less fibrosis, showed a comparatively lower MMP-2 signal (Fig. 13, panel C).

**Fig. 14:**
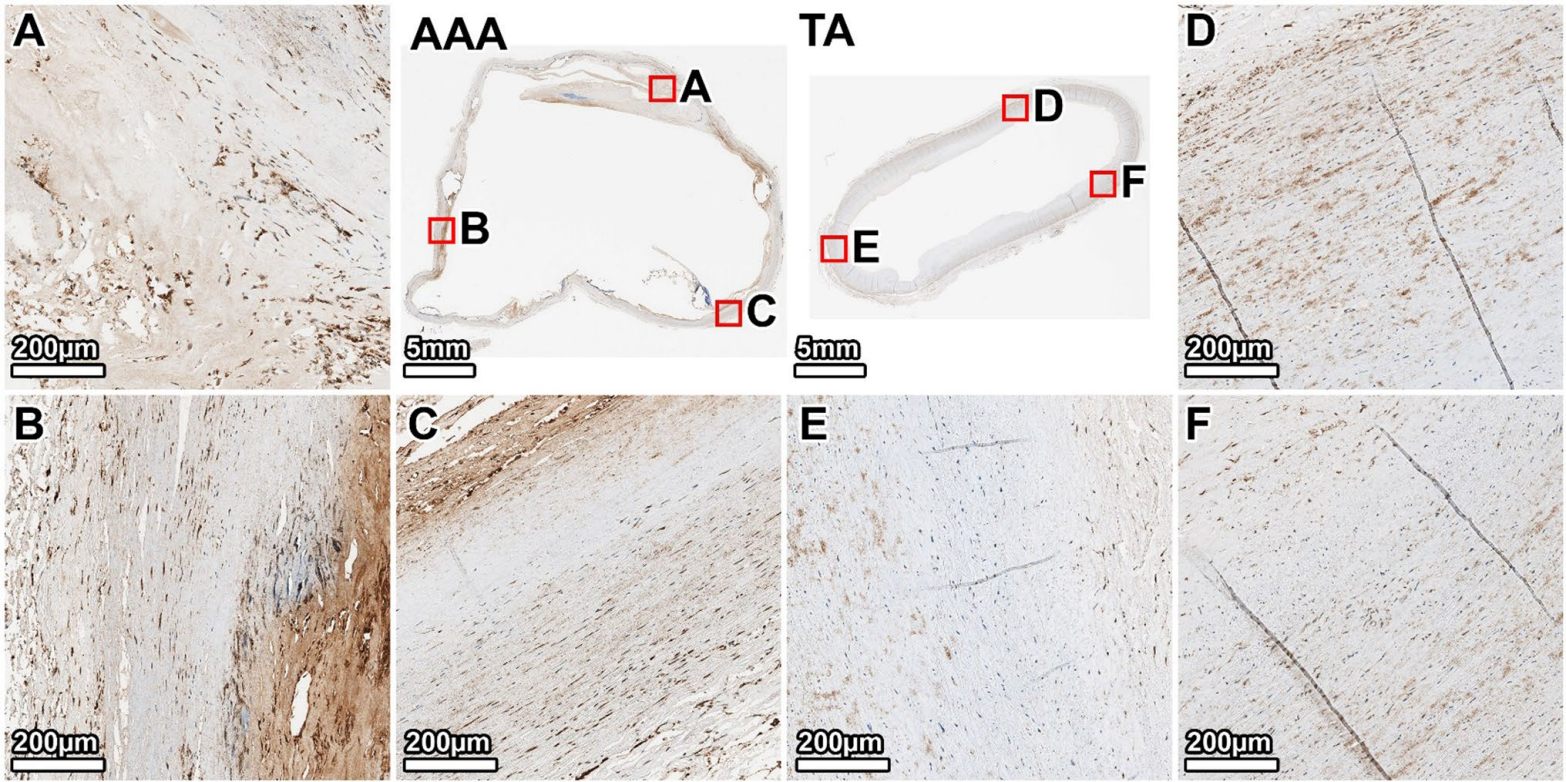
MMP-9 expression in the 89-year-old donor. Staining for MMP-9 is minimal in the TA. In the AAA, expression is elevated and concentrated in the media of the most fibrotic regions (left and right anterolateral walls). Panels show whole-section views of the AAA and TA rings, with high-magnification views from the posterior (**A**, **D**), right anterolateral (**B**, **E**), and left anterolateral (**C**, **F**) walls

MMP-9 expression was very low in the TA of the 89-year-old donor (Fig. 14, panels D–F). In the AAA, the signal was diffuse but clearly elevated compared to the TA. MMP-9 expression was most prominent in the tunica media of the heavily fibrotic posterior and right anterolateral regions (Fig. 14, panels A, B). The less degraded left anterolateral wall showed a weaker MMP-9 signal (Fig. 14, panel C).

#### Broad-spectrum MMP activity

**Fig. 15:**
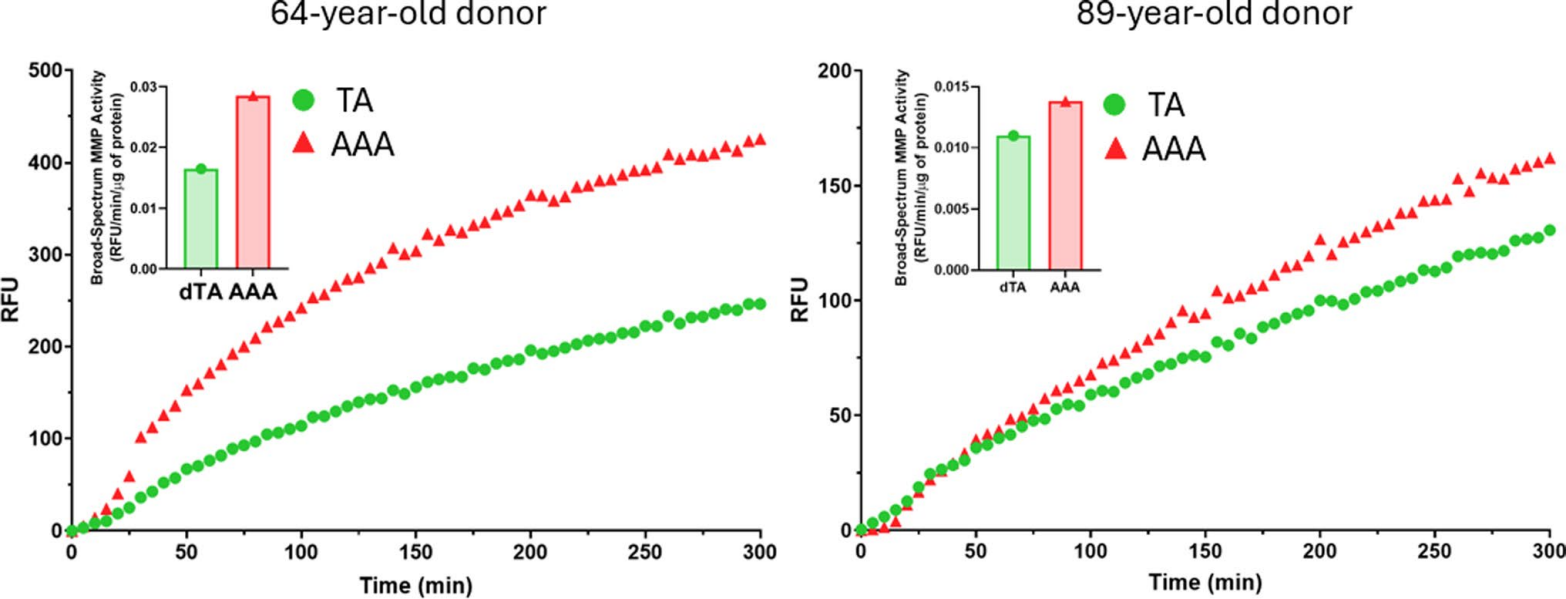
The rate of fluorescence generation, indicating total MMP activity, was measured over time in tissue homogenates from the 64-year-old (left) and 89-year-old (right) donors. In both donors, the AAA tissue (red triangles) exhibited a significantly higher rate of total MMP activity compared to the non-aneurysmal TA (green circles). Inset bar graphs show the mean MMP activity rate (RFU, Relative Fluorescence Units/min/µg of protein)

Broad-spectrum MMP activity on tissue homogenates from the TA and AAA of both donors showed that in both cases, the total MMP activity was significantly higher in the AAA tissue compared to the corresponding TA tissue (Fig. 15). For the 64-year-old donor, the rate of enzymatic activity in the AAA was nearly double that of the TA. A similar, though less pronounced, increase was observed in the 89-year-old donor, confirming elevated proteolytic activity within the aneurysmal wall.

#### Gelatin zymography for MMP-2 and MMP-9 activity

**Fig. 16:**
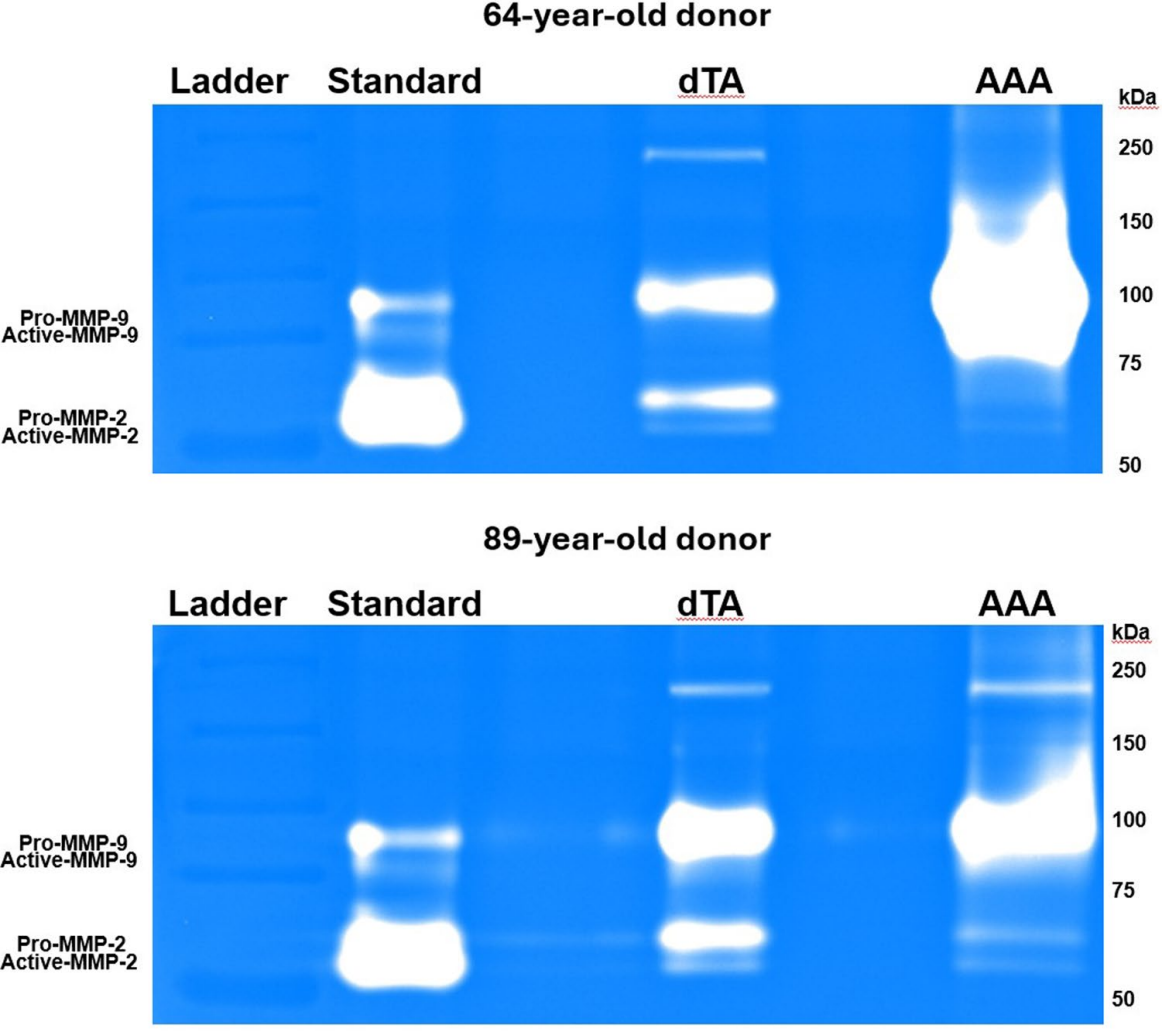
Gelatin zymography of MMP-2 and MMP-9 activity. Gelatinolytic activity in tissue extracts from the TA and AAA of the 64-year-old (top) and 89-year-old (bottom) donors. The analysis revealed a significant increase in both pro- and active-MMP-9 activity and a marked decrease in pro- and active-MMP-2 activity in the AAA compared to the TA tissues

Gelatin zymography was used to specifically assess the activity of the gelatinases, MMP-2 and MMP-9. The results revealed a differential regulation of these two enzymes in the aneurysmal tissue (Fig. 16). In both the 64-year-old and 89-year-old donors, the AAA tissue showed a dramatic increase in the activity of both pro- and active-MMP-9 compared to the TA. Conversely, the activity of both pro- and active-MMP-2 was markedly decreased in the AAA tissue of both donors when compared to their respective TA samples.

### Mechanical properties

**Fig. 17:**
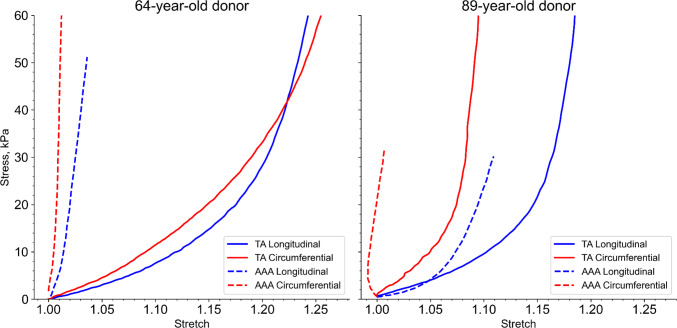
Biaxial mechanical properties of TA and AAA tissues. Equibiaxial stress–stretch curves for tissues from the 64-year-old (left) and 89-year-old (right) donors. The non-aneurysmal TA (solid lines) shows compliant, nonlinear behavior, while the AAA tissue (dashed lines) is stiffer and in both longitudinal (blue) and circumferential (red) directions

Planar biaxial mechanical testing showed significant differences in the material properties between the non-aneurysmal TA and the aneurysmal AAA tissues in both donors (Fig. 17). To quantify these differences, we analyzed extensibility at low (10 kPa) and high (30 kPa) stress levels and calculated anisotropy as the ratio of circumferential to longitudinal stretch. The TA tissues exhibited the characteristic nonlinear, anisotropic behavior of healthy elastic arteries (Jadidi et al. 2020). In the 64-year-old donor, the TA was relatively isotropic (anisotropy index ~ 0.99), with a similar compliant response in both the circumferential and longitudinal directions reaching stretches of 1.19–1.20 at 30 kPa. In contrast, the TA from the 89-year-old donor was stiffer overall (longitudinal stretch = 1.16, circumferential stretch = 1.08 at 30 kPa) and exhibited moderate anisotropy (anisotropy index 0.93), consistent with age-related changes, and was more compliant in the longitudinal direction. For both donors, the AAA tissue was dramatically stiffer and significantly less extensible in both directions compared to the TA. This was most pronounced in the 64-year-old AAA, where the tissue behaved as a near-rigid tube with negligible extensibility, reaching stretches of only 1.01–1.03 even at high stress (30 kPa). In the 89-year-old donor, this stiffening was particularly more pronounced in the circumferential direction (stretch = 1.01) than longitudinal direction (stretch = 1.11), resulting in a highly anisotropic response (anisotropy index 0.91).

## Discussion

A challenge in the study of AAA pathophysiology has been the confounding influence of inter-subject variability. The majority of previous investigations have relied on comparing aneurysmal tissue from one cohort of patients with healthy aortic tissue obtained from a separate group of organ donors (Annabi et al. 2002; Geest et al. 2004; Vande Geest et al. 2006; Molacek et al. 2014; Niestrawska et al. 2016). This approach, while providing valuable information about AAA, inherently introduces a multitude of uncontrolled variables, including genetic predispositions, comorbidities, lifestyle factors, and baseline differences in aortic architecture. The present study was designed to overcome this limitation by multimodal analysis of the AAA and the non-aneurysmal TA samples from the same individual.

The AAA segments showed severe degeneration of the medial structure, marked elastin loss and SMCs’ depletion, with concomitant fibrosis and calcification, whereas the subject-matched TA segments retained organized lamellar architecture. These structural differences were reflected functionally, with AAA tissue being significantly stiffer and less extensible than the TA. In parallel, enzymatic assays demonstrated a substantially elevated proteolytic activity in AAA walls, particularly driven by increased MMP activity. Taken together, these results highlight the interplay between histopathological degeneration, biochemical proteolysis, and mechanical dysfunction in AAA, while underscoring the value of intra-subject controls in AAA research.

Our histological observations confirm classic hallmarks of AAA and illustrate their extreme manifestation in end-stage disease. In both donors, the abdominal aneurysm wall had degraded elastic lamellae to a far greater degree than in the thoracic aorta, where elastic fibers were intact, aside from mild age-related thinning in the older donor (Fritze et al. 2011; Jadidi et al. 2020; Heinz 2021). This aligns with the well-established description of AAA as a disease of medial elastin destruction and SMCs loss (Campa et al. 1987; López-Candales et al. 1997; Henderson et al. 1999; Wassef et al. 2001; Ailawadi et al. 2003; Guo et al. 2006; Bruijn et al. 2021). Movat’s and trichrome stains revealed dense collagen deposition and glycosaminoglycan accumulation throughout the AAA media, effectively replacing the lost elastin and SMCs structure. Such extensive fibrosis is characteristic of advanced AAA and represents the vessel’s attempt to reinforce the wall after elastin breakdown. Prior studies have similarly noted increased collagen content and scar-like remodeling in AAA tissue (Rizzo et al. 1989; McMahon et al. 1990; He and Roach 1994). In our samples, this fibrotic remodeling was heterogeneous around the circumference, but in all regions, the elastin-to-collagen ratio was profoundly lower in AAA than in the TA. This shift in matrix composition, i.e., loss of elastic fibers and excess collagen/GAG, is known to make the tissue stiffer and less compliant (He and Roach 1994; Niestrawska et al. 2016), consistent with our findings from biaxial mechanical tests.

Our transverse histology demonstrated regional heterogeneity within the aneurysm wall, which also has been reported previously (Menges et al. 2023). In each donor, certain quadrants of the AAA were more severely degraded than others. In the 89-year-old donor, we found that the posterior and right-lateral walls were transformed into collagenous, cell-sparse tissue, whereas the left anterolateral wall preserved more elastic lamellae and SMCs. This non-uniformity appears to correlate with the distribution of the ILT and localized hemodynamic factors. In the 64-year-old donor, for example, the anterior wall of the AAA (adjacent to a large mural thrombus) showed near-total elastin loss and SMC depletion, whereas the posterior wall, which lacked significant thrombus coverage, retained slightly more medial structure. This pattern is consistent with previous reports that the presence of ILT exacerbates underlying wall degeneration (Koole et al. 2013; Ducas et al. 2020; Boyd 2021). ILT-covered regions of AAA wall tend to be thinner, with fewer intact elastin fibers and SMCs and more inflammation, compared to wall areas exposed directly to blood flow (Kazi et al. 2003). Kazi et al. observed that the AAA wall beneath thrombus is infiltrated by inflammatory cells and has a high rate of SMC apoptosis, suggesting the thrombus creates a hostile microenvironment of hypoxia and proteolytic activity that accelerates tissue breakdown (Kazi et al. 2003). These results show that within an aneurysm, one can observe a spectrum from relatively moderate remodeling to end-stage degeneration. This heterogeneity suggests that AAA wall strength is not uniform and the weakest point, which is likely the site of rupture, is often where the wall is most severely affected and thrombosis is present (da Silva et al. 2000). It also underlines the need for spatially resolved assessments of AAA wall health rather than treating the aneurysm as a homogeneous sample.

Our biochemical analyses shed light on the proteolytic profile of AAA tissue, particularly regarding MMP-2 and MMP-9, which are central to AAA pathophysiology (Longo et al. 2002). Total MMP activity from broad-spectrum assays was significantly higher in the AAA segments than in the subject-matched TAs, confirming that aneurysm walls exist in a state of elevated proteolytic stress. This is in agreement with the consensus that MMPs are upregulated in AAA and drive matrix degradation (Annabi et al. 2002; Longo et al. 2002). Immunohistochemistry localized this enzymatic activity to the sites of tissue remodeling. We found intense MMP-2 and MMP-9 staining within the aneurysmal media, especially in regions adjacent to the thrombus or in the most fibrotic areas. Notably, in the 64-year-old donor AAA, MMP-2 was highly expressed at the interface of the ILT and wall, while MMP-9 was more diffusely present in the degenerated media. In the 89-year-old’s AAA, we saw MMP-2 co-localized with α-SMA-positive myofibroblastic cells in heavily scarred regions, and MMP-9 concentrated in those same regions where inflammation was likely most active.

The fact that MMP-9 staining was elevated in our AAA tissues is expected, as MMP-9 is the most abundantly expressed elastolytic MMP in human AAA (Thompson et al. 1995; Longo et al. 2002). MMP-9’s presence in high levels corresponds with the extensive elastin fragmentation we observed. Active-MMP-9 can degrade elastic fibers and has been implicated in driving aneurysm expansion (Petrinec et al. 1996). Prior studies have linked high MMP-9 levels with more rapidly growing or ruptured aneurysms (Petersen et al. 2000; Ailawadi et al. 2003), underlining its significance as both a mediator and potential marker of aggressive AAA disease.

One intriguing finding in our study was the differential activity of MMP-2 in AAA versus control. Although MMP-2 immunostaining was elevated in AAA tissue, zymography revealed reduced MMP-2 activity compared with TA. This discrepancy likely reflects the fact that IHC measures total protein abundance, including inactive pro-MMP-2, whereas zymography specifically measures enzymatic activity. Elevated immunostaining likely indicates the accumulation of inactive protein trapped within the extensive fibrotic matrix, rather than functionally active enzyme. At first glance, this seems contradictory to reports of increased MMP-2 in AAA tissue relative to healthy aortas (Davis et al. 1998; Goodall et al. 2001; Longo et al. 2002). However, our findings are not unprecedented. It likely reflects the complex regulation and stage-dependent role of MMP-2 in AAA. Prior studies noted that while the pro-MMP-2 level was reduced in AAA tissue, the fraction of MMP-2 in the active form was unchanged (Annabi et al. 2002). It is important to consider that MMP-2 is constitutively produced by SMCs in the aortic wall. In early aneurysm development, as SMCs become activated or as fibroblasts infiltrate, MMP-2 production and activation can increase (Ailawadi et al. 2003). Indeed, high MMP-2 concentrations have been observed in small early stage AAAs (Freestone et al. 1995; Ailawadi et al. 2003), supporting a role for MMP-2 in the initial elastin degradation that initiates aneurysm formation. By the time aneurysms are large and advanced, as in our donors, the medial SMC population is widely depleted. Since these cells are the primary source of MMP-2, their loss suggests that the capacity for generating new, active enzyme is halted. This depletion of the cellular source could explain the lower activity levels observed on zymography compared to the healthy TA, where SMCs remain viable and productive.

In contrast, MMP-9 activity was strongly elevated in the AAA, as our zymography showed, a finding consistent with prior studies (Thompson et al. 1995; Sakalihasan et al. 1996; Yamashita et al. 2001). Taken together, these results paint a picture in which MMP-9 is the dominant gelatinase in late-stage AAA (Petersen et al. 2000), while MMP-2’s role may peak earlier and then taper off as the cellular source is lost (Freestone et al. 1995). The concerted action of these MMPs over the course of the disease leads to progressive matrix degradation. MMP-2 likely contributes to early elastin damage and weakening of the wall, and MMP-9 propagates ongoing proteolysis and tissue turnover in the expanding aneurysm.

Micro-CT imaging allowed us to visualize and quantify calcific deposits in the aortas, revealing a substantial burden of calcification in the aneurysmal segments. Both donors’ AAAs contained a mixture of sheet-like calcifications and nodular discrete calcifications, whereas the thoracic aorta controls had only sparse or smaller calcific plaques. This confirms that calcification is a frequent component of AAA pathology. It has been reported that the majority of AAAs have calcified regions (Buijs et al. 2013; Chowdhury et al. 2018), ranging from small microcalcifications to large, plate-like deposits (Basalyga et al. 2004; Kuntz et al. 2019; Li et al. 2020; Bruijn et al. 2021). In our younger donor’s aorta, calcification volume approximately doubled within the aneurysm compared to the adjacent normal aorta segment, and in the older donor, an even more dramatic calcification increase was seen distally as the aneurysm extended toward the iliac arteries. The presence of extensive calcification in AAA tissue has important implications for biomechanics, and calcification generally makes the arterial wall less compliant.

The biaxial mechanical tests demonstrated how all these tissue changes, including elastin loss, fibrosis, and calcification, translate into altered material behavior. Aneurysmal samples from both donors were markedly stiffer and less extensible in both circumferential and longitudinal directions compared to the respective thoracic controls. The TA segments behaved like normal elastic arteries, with nonlinear and compliant stress–stretch response, especially the 64-year-old’s TA (Jadidi et al. 2020, 2021). In contrast, the AAA segments showed a stiffer stress–stretch response, with lower stretch values at different stress levels compared to TA. It is well documented that AAA walls are mechanically stiffer and less distensible than healthy aortas (He and Roach 1994; Vorp et al. 1996; Vande Geest et al. 2006; Vorp 2007). Our multimodal analysis shows that the loss of elastin and SMCs, and the increase in collagen and calcifications, in AAA fundamentally shifts the wall’s material properties. It is important to note, however, that the macroscopic stiffening observed in our biaxial tests reflects the composite structural alterations of the vessel wall. While micro-CT confirmed significant calcification and histology revealed extensive fibrosis, our current methodology cannot quantitatively decouple the specific mechanical contribution of calcification from that of matrix remodeling. Future studies utilizing microscale techniques, such as atomic force microscopy or nanoindentation, are necessary to differentiate the localized stiffening effects of calcification versus fibrosis. We also noted that the older donor’s tissues were generally less compliant than the younger donor’s, even in the thoracic aorta, confirming that aging itself stiffens the aorta (Jadidi et al. 2020, 2021).

While these results provide a comprehensive picture of the AAA pathophysiology, they need to be considered within the study limitations. The primary limitation of this study is the small sample size, with only two donors analyzed. While the intra-subject design substantially reduces variability and provides unique insights into AAA pathology, acquiring intact aortas encompassing the entire thoracic and abdominal segments with untreated aneurysms is logistically challenging. Unlike studies utilizing surgical biopsies, this design requires whole aortas which are rare. Consequently, our findings should be interpreted as descriptive case studies rather than broadly generalizable. In addition, both donors were female, which does not reflect the higher prevalence of AAA in males. Future efforts will focus on expanding this biobank, including male donors, and investigating sex as a biological variable. Post-mortem tissue handling and freezing, though carefully controlled, may also introduce artifacts that differ from in vivo physiology. Furthermore, it should be acknowledged that even in healthy aortas, there is inherent variation in structure and mechanics along the length and circumference of the vessel, and comparisons between thoracic and abdominal segments, while informative, are not without limitations (Kazim et al. 2024; Shahbad et al. 2025). Additionally, we did not perform precise spatial co-registration between the micro-CT data, the mechanical testing sites, specimens for MMP activity, and histology sections. Performing such co-registration requires time to reconstruct and map the images, which would have delayed tissue processing and likely caused MMP degradation, thereby affecting the accuracy of our enzymatic assays. Similarly, we could not correlate regional mechanical properties with specific histological quadrants due to geometric constraints (transverse rings for histology versus square samples for biaxial testing). Future investigations could address this by performing histological analysis directly on the mechanically tested specimens. Finally, while our multimodal approach integrated histology, imaging, biochemistry, and mechanics, other important contributors to AAA progression, such as inflammatory cytokines, genetic predisposition, and hemodynamic stresses, were not directly assessed. These limitations highlight the need for future studies with larger and more diverse cohorts, as well as incorporation of additional molecular and biomechanical analyses.

## Conclusion

**Fig. 18:**
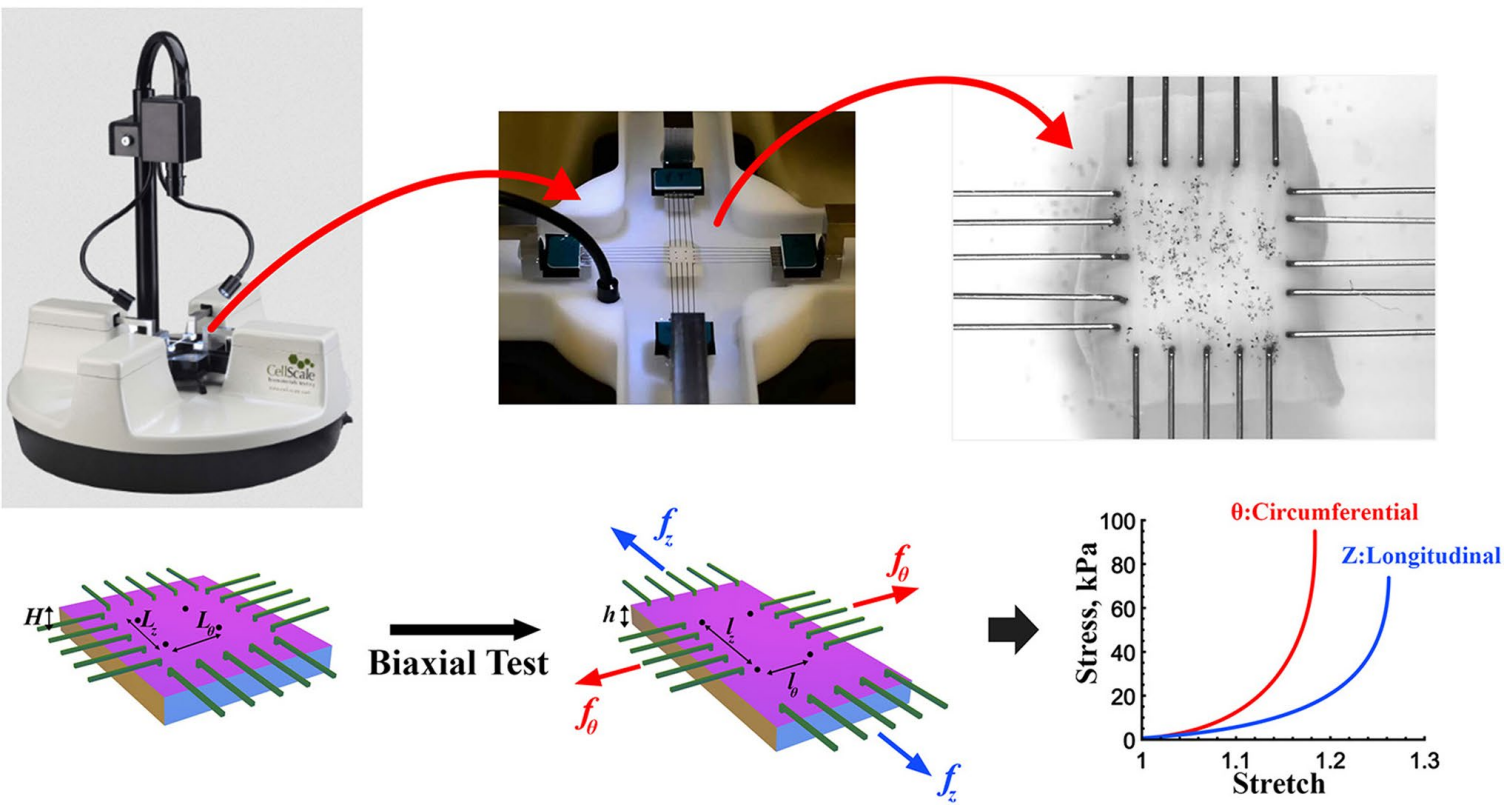
A representative setup of the CellScale planar biaxial testing system (left), specimen mounted with rakes, and magnified view of the central region of interest with graphite particles used for digital image-based deformation tracking (right). Schematic illustrates the square tissue specimen subjected to controlled tensile loading in the circumferential (*θ*) and longitudinal (*z*) directions, with corresponding forces, initial gauge lengths, and stretch directions. Equibiaxial loading produces stress–stretch responses in both directions, shown by representative nonlinear curves for circumferential (red) and longitudinal (blue) behavior. This configuration was used to quantify passive anisotropic mechanical properties of thoracic and aneurysmal aortic tissues

In summary, this multimodal, intra-subject analysis provides a comprehensive characterization of AAA pathology, demonstrating the interconnected roles of elastin degradation, smooth muscle cell loss, fibrosis, calcification, and heightened MMP activity in driving aneurysmal degeneration and mechanical stiffening. Despite its limited sample size, this study underscores the value of subject-matched controls in disentangling disease-specific changes and offers a framework for future investigations into the pathophysiology of AAA (See Fig. 18).

## Data Availability

Research data supporting the findings of this study are available from the corresponding author upon reasonable request.

## Appendix

See Fig. 18.
